# Optimizing photosynthetic light-harvesting under stars: simple and general antenna models

**DOI:** 10.1007/s11120-024-01118-1

**Published:** 2024-09-10

**Authors:** Samir Chitnavis, Callum Gray, Ifigeneia Rousouli, Edward Gillen, Conrad W. Mullineaux, Thomas J. Haworth, Christopher D. P. Duffy

**Affiliations:** 1 School of Biological and Behavioural Sciences, Queen Mary University of London, Mile End, London, E1 4NS UK; 2 Digital Environment Research Institute, Queen Mary University of London, Empire House Whitechapel, London, E1 1HH UK; 3 Astronomy Unit, Queen Mary University of London, Mile End Road, London, E1 4NS UK

**Keywords:** Light-harvesting, Antennae, Cyanobacteria, Thermodynamic models, Astrobiology

## Abstract

In the next 10–20 years, several observatories will aim to detect the signatures of oxygenic photosynthesis on exoplanets, though targets must be carefully selected. Most known *potentially habitable* exo-planets orbit cool M-dwarf stars, which have limited emission in the photosynthetically active region of the spectrum (PAR, $400< \lambda < 700$ nm) used by Earth’s oxygenic photoautotrophs. Still, recent experiments have shown that model cyanobacteria, algae, and non-vascular plants grow comfortably under simulated M-dwarf light, though vascular plants struggle. Here, we hypothesize that this is partly due to the different ways they harvest light, reflecting some general *rule* that determines how photosynthetic antenna structures may evolve under different stars. We construct a simple thermodynamic model of an oxygenic antenna-reaction centre supercomplex and determine the optimum structure, size and absorption spectrum under light from several star types. For the hotter G (e.g. the Sun) and K-stars, a small modular antenna is optimal and qualitatively resembles the PSII-LHCII supercomplex of higher plants. For the cooler M-dwarfs, a very large antenna with a steep ’energy funnel’ is required, resembling the cyanobacterial phycobilisome. For the coolest M-dwarfs an upper limit is reached, where increasing antenna size further is subject to steep diminishing returns in photosynthetic output. We conclude that G- and K-stars could support a range of niches for oxygenic photo-autotrophs, including high-light adapted canopy vegetation that may generate detectable bio-signatures. M-dwarfs may only be able to support low light-adapted organisms that have to invest considerable resources in maintaining a large antenna. This may negatively impact global coverage and therefore detectability.

**Supplementary Information:**

The online version contains supplementary material available at 10.1007/s11120-024-01118-1.

## Introduction

**Fig. 1:**
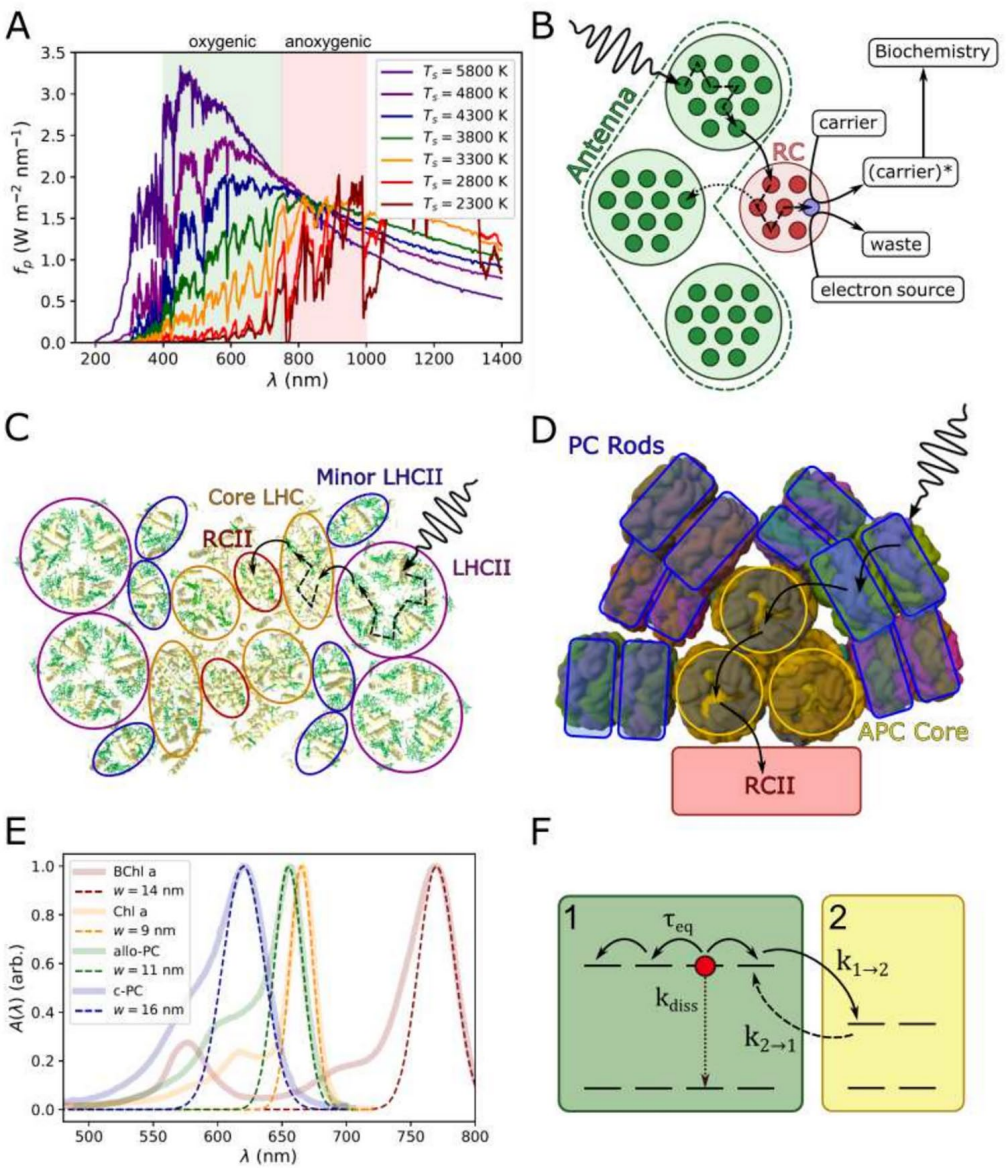
**A** Incident spectral fluxes, $f_{p}\left( \lambda ;T_{s},a_{\text {sp}}\right)$ for a range of stellar temperatures, $T_{s}$. The green and red shaded boxes indicate the approximate absorption windows for oxygenic (photosynthetically active radiation, PAR) and anoxygenic photosynthesis respectively; the red and orange plots are representative of M-dwarf stars while the purple plot is representative of the Sun. **B** Schematic of the generalized photosystem in which a RC (red) is fed excitons by an antenna composed of multiple light-harvesting proteins (green). Energy rapidly equilibrates within an LHC subunit (dashed) arrows and then hops to another complex (solid arrows). Once on the RC it can reach the trap (blue) where the energy is used to oxidize an electron source and reduce an electron carrier. **C** A schematic of the PSII supercomplex from plants as it lies in the plane of the membrane (adapted from PDB: 5XNM (Su et al. 2017)). The antenna is composed of different chlorophyll-binding LHC sub-units (LHCII and minor variants plus the ‘core antenna’ complexes). The PSII RC (labelled RCII is shown in red). **D** schematic of the bilin-binding phycobilisome antenna of the cyanobacterium *Synechococcus* sp. PCC 7002, as viewed along the plane of the membrane (adapted from PDB: 7EXT (Zheng et al. 2021)). Phycocyanin (PC, blue) sub-units are stacked into branches or rods which radiate out from an antenna core of allophycocyanin (APC, yellow) sub-units. These are connected to essentially the same RCII complex found in plants. **E.** The lowest energy absorption bands of photosynthetic pigments Bacteriochlorophyll *a* (red), Chlorophyll *a* (orange), allophycocyanin (green) and c-phycocyanin (blue). The dashed lines represent Gaussian fits with widths in the range $w=9-16$ nm. Note that we do not fit the blue vibronic edge of each pigment, which is quite pronounced for c-PC. **F.** Schematic of energy hopping between two LHC sub-units (labelled 1 and 2). Here forward transfer, $k_1\rightarrow _2$, is *enthalpically* favourable since it involves a reduction in excitation energy. However, it is *entropically* unfavourable since the excitation will have fewer pigments to sample in the smaller sub-unit 2

At time of writing roughly 5500 extra-solar planets (exo-planets) have been identified (NASA Exoplanet Archive 2024). Of these, approximately 100 are deemed potentially habitable (Arecibo 2024; Méndez et al. 2021; Meadows and Barnes 2018; McKay 2014), though most of these orbit M-dwarf stars, stars that are significantly cooler and redder than the Sun. These include the planetary systems of TRAPPIST-1 (Gillon et al. 2017; Grimm et al. 2018), Proxima Centauri (Anglada-Escudé et al. 2016; Faria et al. 2022) and LHS 1140 (Dittmann et al. 2017), which are frequently discussed in terms of extra-solar life. M-dwarf planets are generally considered good astrobiological targets for the following reasons. Stellar formation models predict that planets occur more frequently around M-dwarfs than around larger/hotter stars (Mulders et al. 2015; Hsu et al. 2019, 2020). Moreover, the stable phase of their lifespan (the *main sequence lifetime*) lasts billions of years, meaning that complex biospheres would have time to evolve (Laughlin et al., 1997; Shields et al., 2016). Additionally, potentially habitable planets reside close to the star leading to more robust characterisation of their atmospheres (Shields et al. 2016). However, one downside is that, due to their low temperature ($T_{s}\sim 2300$ K), M-dwarf stars have very limited emissions in the region of the spectrum ($400< \lambda < 700$ nm) traditionally considered as *photosynthetically active radiation* (PAR) (shown in Fig. 1A).

A biosphere requires energy input, with the parent star being the most obvious candidate (of course geothermal (Dodd et al. 2017) and geochemical (Ricci and Greening 2023) energy sources may also contribute), meaning it is reasonable to assume that exo-planetary biospheres will be founded on some form of photosynthesis. Oxygenic photosynthesis in particular is the focus of much astrobiological research for several reasons. It may have been a necessary prerequisite for the evolution of multicellular life on Earth (though there is considerable debate (e.g. Wood et al. 2020; Mills and Canfield 2014; Butterfield 2009; Cole et al. 2020; Bozdag et al. 2021), meaning it is a required basis for diverse and complex biospheres. More importantly, it may present the best chance of actually detecting an exo-biosphere in the near future (Kiang 2014), either via detection of $O_3$ in atmospheric transmission spectra (Mendillo et al. 2018; Olson et al. 2018a, b; Lyons et al. 2014; Schwieterman et al. 2018), or by a strong depletion in the range 400–700 nm, in a surface reflectance signal from the planet (O’Malley-James and Kaltenegger 2018; Seager et al. 2005; Arnold et al. 2002; Battistuzzi et al. 2020). The latter is known as a *vegetation red edge* (VRE) and was first observed for Earth by the Galileo space probe (Sagan et al. 1993), an unmistakable signature of widespread, chlorophyll-rich vegetation. The question of where we should and shouldn’t look for signs of oxygenic photosynthesis is serious one. The James Webb Space Telescope (JWST) and the upcoming (2029) Ariel mission (Tinetti et al. 2020) will study the molecular composition of exo-planet atmospheres. Planned (next 10–20 years) observatories such a the Extremely Large Telescope (Bowens et al. 2021), the Habitable Worlds Observatory (Gaudi et al. 2019), and the Large Interferometer For Exoplanets (Quanz et al. 2022) will measure reflected light from the surface of rocky exo-planets. Of course, the huge resource investment, large measurement time and finite instrument lifetimes mean that candidate planets should be carefully targeted. This requires some method of ranking potential targets in terms of their potential to harbour *detectable* life based on bulk properties of the planet and parent star. A key part of this involves understanding the relationship between the feasibility and potential characteristics of photosynthesis and the irradiant spectral flux available in the environment.

The feasibility of oxygenic photosynthesis under M-dwarf light was recently and conclusively demonstrated by the La Rocca group in Padua. Battistuzzi et al. (2023a) showed that cyanobacteria could comfortably grow and produce $O_2$ under simulated M-dwarf light, even with a simulated atmosphere which attenuated the irradiance. This was extended to several eukaryotic macroalgae, the breophyte moss *Physcomitrium patens*, and the model vascular plant *Arabidopsis thaliana*, though the latter showed signs of shade avoidance syndrome (Battistuzzi et al. 2023b). As important as these results are, this does not mean analogues of these organisms would *evolve* in such light environments, with vascular organisms in particular seeming poorly suited. Complementary to this approach are theoretical models that characterize some simple relationships between incident light and photosynthetic strategies. Kiang et al. (2007b) derived an empirical rule-set via an exhaustive review of different organisms. They concluded that photoautotrophs will evolve to have their absorption maximum close to the local irradiance maximum, will evolve secondary pigments that absorb at shorter wavelengths, and will have a reaction centre (RC) that operates close to the red edge of the irradiance window. Applied to M-dwarf light, this implies anoxygenic organisms that absorb in the $930< \lambda < 2500$ nm (Kiang et al. 2007a). Björn (1976) and later Marosvölgyi and van Gorkom (2010) proposed that photosynthetic structures evolve to maximize light absorption while minimising the metabolic cost of synthesizing pigments, neatly predicting the absorption spectra of both higher plants and purple bacteria. Lehmer et al. (2021) applied this model to a range of stellar irradiances, concluding that M-dwarf light would select for organisms that utilize near-infrared ($\lambda \sim 1000$ nm) light. Arp et al. (2020) argued that photosynthetic antennae evolve to be robust against a noisy energy input, which necessarily selects for two sub-populations of pigments with similar (but not identical) absorption maxima, that absorb on the edge of the irradiance window (e.g. Chl *a* and *b* in the case of plants). A modified version of Arp’s principles were applied by Duffy et al. (2023) to various stellar spectra, concluding that M-dwarf stars may preferentially select for anoxygenic photoautotrophs. Lingam et al. (2021) arrived at a similar conclusion based on the *pi*-electron conjugation length that pigments would need to efficiently absorb light in different stellar fluxes. Finally, Hall et al. (2023) considered how photosynthetic feasibility would intersect with *habitability* in general, predicting that oxygenic photosynthesis may be restricted to hotter and bluer stars like the Sun. However, a notable feature of these works is that, while they consider the overlap between the light-harvesting antenna and the spectral irradiance, they do not consider the structure, size and overall efficiency.

Oxygenic photosynthesis can and does exist in niches on Earth with very limited PAR, due to the evolution of the antenna-RC architecture (Wolfe et al. 1994; Fleming et al. 2012). While there is considerable diversity in antenna structures, they all function in the same way: a large, modular assembly of pigment-protein complexes captures light and directs the resulting excitation energy into a much smaller central RC (sketched in Fig. 1B). They are generally also adaptable structures, with antenna size changing as organisms acclimate to high or low light, maximizing light input in the latter and mitigating photo-damage in the former (Sanfilippo et al. 2019; Lokstein et al. 2021). Still, there are differences between how plants and cyanobacteria harvest light, which may be relevant to why plants struggle in M-dwarf light and cyanobacteria appear to thrive (Battistuzzi et al. 2023b). Photosystem II of higher plants (PSII, the oxygen-producing photosystem) has a transmembrane antenna composed of structurally similar antenna sub-units binding energetically similar pigments (Su et al. 2017). The supercomplex formed of the PSII RC core (RCII), several minor/monomeric LHCII antenna complexes and several bound trimeric LHCII, sit in a wider, disordered pool of peripheral LHCII (see Fig. 1C). It is this modularity that enables plant PSII to rapidly adapt to changes in the light environment (Vialet-Chabrand et al. 2017; Ruban 2016; Ruban and Wilson 2021). Cyanobacteria and some red algae possess the phycobilisome (PBS) antenna which sits out of the plane of the membrane and has a hierarchical rather than modular structure. A core of allophycocyanin (APC) proteins form a hub from which rods of bluer phycocyanin (PC) and phycoerythrin (PE) radiate (Zheng et al. (2021), see Fig. 1D). How do these two light-harvesting systems *fundamentally* differ? Is one more likely to evolve under M-dwarf light than the other?

In this work we construct a simple and general model of a RC-antenna light-harvesting system. It is simple in the sense that it considers only the basic thermodynamics of light-harvesting, and it is general in the sense that it presupposes no (or very little) molecular detail. Our aim is to predict what type of antenna structures are best suited to a range of irradiant fluxes from a range of different star types. The null hypothesis is that, so long there is some flux in the PAR region, a sufficiently large antenna will facilitate oxygenic photosynthesis. The alternative hypothesis is that oxygenic photosynthesis under an M-dwarf star will *require* a more hierarchical (PBS-like) antenna than hotter stars. Harvesting light, that is, absorbing photons over a large area and concentrating that energy into a small RC, is a process that reduces *entropy* (see Fig . 1 F). This results in steeply diminishing returns in light-harvesting efficiency with increasing antenna size. To overcome this and make light-harvesting thermodynamically favourable (or less unfavourable) antennae adopt, to greater or lesser degrees, an enthalpy funnel structure, with excitations transferred from higher to lower-energy pigments. We argue that the fundamental difference between the cyanobacterial PBS and the plant PSII antenna is how and to what extent they compensate for this entropy penalty. The former has a steeper funnel than the later, along with multiple independent antenna *branches*. We show that the PBS is inherently much more adaptable to the type of limited PAR light produced by a M-dwarf star.

## Methods

### A simple and general model of a photosynthetic antenna-RC system

A generalized photosynthetic system (hereafter “photosystem”) is sketched in Fig. 1B and has the following properties:

1. A central RC that carries out a general photo-chemical reaction, oxidizing an electron donor and then reducing an electron carrier. The energy required to oxidize the donor sets the peak absorption wavelength of the RC, ${\lambda }_{{p}}^{{r}}$.
2. The antenna is an assembly of $N_{\text {LHC}}$ LHC sub-units which which harvest light and transfer excitation energy to the RC. The *i*th LHC has peak absoprtion wavelength, $\lambda _i^p$.
3. The RC and LHCs bind organic, $\pi$-conjugated pigment molecules. We will assume these have an approximately Gaussian absorption spectrum centred on $\lambda _i^p$, with a standard deviation (hereafter the ‘width’) of the order ${w}\sim {10}$ nm (see Fig. 1E for a selection of pigment absorption spectra taken from Taniguchi and Lindsey 2018). The optical/absorption cross-section of such pigments is of the order ${\sigma } = {10}^{{-21}}-{10}^{{20}}$ m$^{{2}}$ (Ye et al. 2023).
4. Photon absorption in the antenna creates a localized excitation (exciton) that diffuses through the antenna to the RC. The characteristic timescale for excitation ‘hopping’ between sub-units is of the order ${k}_{\text {hop}}^{{-1}}\sim 10$ ps (Novoderezhkin et al. 2011; Valkunas et al. 2009). Excitation equilibration within an LHC sub-unit is assumed to be much faster, ${\tau }_{{i}}^{{eq}}\rightarrow {0}$.
5. The RC and LHCs contain on the order of 10–100 pigments. Equilibration within larger ($>>100$ pigments) sub-units becomes diffusion-limited and is better represented in our model using a block of several identical sub-units.
6. Evolution will select for pigments with long excitation lifetimes (of the order ${k}_{{{diss}}}^{-1} \sim {1}$ ns) so that excitation decay (dissipation) does not out-compete energy transfer to the RC, ${k}_{{i\rightarrow j}} \gg {k}_{{{diss}}}$.
7. The RC sub-unit contains an irreversible ‘trap’ which converts the exciton into a *charge-separated state*. The kinetics of trapping are *at least* as fast as inter-sub-unit energy transfer, ${k_{{{trap}}}} \ge {k_{\text {hop}}}$ (Broess et al. 2006).
8. The electron acceptor is reduced by the charge-separated state and the now oxidized $\hbox {RC}^+$ is reduced (effectively re-set) by the electron donor (e.g. water, H$_2$O). The kinetics of this cycle define the *electron output rate* which is assigned a rate constant, ${k_{\text {out}}}$. The RC can only process one excitation at a time.

A generalized *oxygenic* photosystem has the following additional properties:

1. The RC oxidizes H$_2$O, requiring ${\lambda }^{{r}}_{{p}}\sim {680}$ nm.
2. The kinetics of H$_2$O oxidation are rate-limiting with a rate constant of order ${k}_{{{out}}}^{{-1}} \sim {10}$ ms (Oja and Laisk 2000).

### Local spectral irradiance as a function of stellar temperature

As in previous work (Duffy et al. 2023), we use stellar spectral models for stars of different effective temperatures, $T_{s}$, generated by the phoenix code (Husser et al. 2013). To reduce expense of subsequent numerical integration calculations, we smooth and re-sample the spectrum down to 4000 points, which still captures the large scale features. The absorption profiles of typical light-harvesting pigments are quite broad, with a *width* $\sim 10$ nm, meaning that the photosynthetic pigments can only *resolve* spectral details on this order. Even with this smoothing, spectral resolution is well below this limit. We relate the stellar surface spectral flux density, $I_{s}\left( \lambda ;R_{s},T_{s}\right)$ to the spectral flux density at the planetary surface, $f_{p}\left( \lambda ;R_{s},T_{s},a_{\text {sp}}\right)$, with the radiative equilibrium condition,

1 $$\begin{aligned} f_{p}\left( \lambda ;R_{s},T_{s},a_{\text {sp}}\right) = \left( \frac{R_{s}}{a_{\text {sp}}}\right) ^{2} I_{s}\left( \lambda ;R_{s},T_{s}\right) \end{aligned}$$

where $R_{s}$ is the stellar radius, $a_{\text {sp}}$ is the mean orbital distance of the planet and both $I_{s}\left( \lambda \right)$ and $f_{p}\left( \lambda \right)$ have units of W $\hbox {m}^{-2}$ $\hbox {nm}^{-1}$. During the stellar main sequence $R_{s}$ and $T_{s}$ are related by a power law and and therefore we hereafter specify $I_{s}(\lambda ;T_{s})$ and $f_{p}\left( \lambda ;T_{s},a_{\text {sp}}\right)$ in terms of $T_{s}$ only. We consider $2300 K \le T_{s} \le 5800$ K , with $T_{s} = 2300$ K representing the coolest end of the M-dwarf stellar range and $T_{s} = 5800$ K giving an approximation of the spectral flux density of the Sun. We obtain approximate habitable orbital distances using the relation,

2 $$\begin{aligned} a_{\text {sp}} = \frac{R_{s}}{2} \left( \frac{T_{s}}{T_{p}} \right) ^{2} \end{aligned}$$

where $T_{p}$ is the average planetary surface temperature, taking the mid-point between the values of $a_{\text {sp}}$ that yield $T_{p}=273$ K (‘ice line’) and 373 K (‘steam line’). Note, that for reasons of generality we do not consider any albedo (reflectance) or atmospheric attenuation of $f_{p}\left( \lambda ;T_{s},a_{\text {sp}}\right)$.

### Modelling the stochastic dynamics of light-capture, energy transfer and electron output

#### The equations of motion

We assume the antenna consists of $N_{\text {LHC}}$ LHC sub-units which each contain $N_i$ identical pigment states. The RC contains $N_r$ pigment states plus a single ($N_t=1$) trap state. The number of excitations within each sub-unit are defined by occupation numbers which can take the following range of values,

3 $$\begin{aligned} n_i&=0,1,2,\ldots ,N_i \end{aligned}$$

4 $$\begin{aligned} n_r&=0,1,2,\ldots ,N_r \end{aligned}$$

5 $$\begin{aligned} n_t&=0,1 \end{aligned}$$

where the full set of occupation numbers at a given time, *t*, defines the instantaneous *configuration* of supercomplex. We define the joint, time-dependent probability, $P(n_1,n_2,\ldots ,n_i,\ldots ,n_{N_{LHC}},\dots ,n_r,n_t,t)$, as the probability of the supercomplex being in a particular configuration at a particular time. We will hereafter use the condensed notation,

6 $$\begin{aligned} P_{n_r,n_t}(t)&=P\left( n_1,n_2,\ldots ,n_i,\dots ,n_{N_\text {LHC}},n_r,n_t,t\right) \end{aligned}$$

7 $$\begin{aligned} P_{n_r,n_t}^{m_i}(t)&=P\left( n_1,n_2,\ldots ,m_i,\dots ,n_{N_{\text {LHC}}},n_r,n_t,t\right) \end{aligned}$$

8 $$\begin{aligned} P_{n_r,n_t}^{m_i,m_j}(t)&=P\left( n_1,n_2,\ldots ,m_i,\dots ,m_j,\dots ,n_{N_{\text {LHC}}},n_r,n_t,t\right) \end{aligned}$$

and so on. The time-evolution of these probabilities is a combination of photon absorption, excitation dissipation, energy transfer, energy trapping by the RC, and the output of electrons via reduction of the electron acceptor,

9 $$\begin{aligned} \begin{aligned} \frac{d}{dt}P_{n_r,n_t}(t)&=\left[ \frac{d}{dt}P_{n_r,n_t}(t)\right] _{abs}+\left[ \frac{d}{dt}P_{n_r,n_t}(t)\right] _{\text {diss}}\\&\quad +\left[ \frac{d}{dt}P_{n_r,n_t}(t)\right] _{\text {trans}}+\left[ \frac{d}{dt}P_{n_r,n_t}(t)\right] _{\text {trap}}\\&\quad +\left[ \frac{d}{dt}P_{n_r,n_t}(t)\right] _{out} \end{aligned} \end{aligned}$$

The kinetics of photon absorption are given by,

10 $$\begin{aligned} \begin{aligned} \left[ \frac{d}{dt}P_{n_r,n_t}(t)\right] _\text {abs}&=-\sum _{i}\gamma _i\left( N_i-n_i\right) P_{n_r,n_t}(t)\\&\quad +\sum _{i}\gamma _i\left( N_i-n_i+1\right) P_{n_r,n_t}^{n_i-1}(t) \end{aligned} \end{aligned}$$

where $\gamma _i$ is the excitation rate of a single pigment belonging to the *i*th LHC. The first term on the right-hand-side (RHS) of Eq. (10) corresponds to all excitation processes,

11 $$\begin{aligned} (n_1,\ldots ,n_i,\ldots ,n_r,n_t)\rightarrow (n_1,\ldots ,n_i+1,\ldots ,n_r,n_t) \end{aligned}$$

with the negative sign implying flow of probability *away* from $P_{n_r,n_t}$ *to* all possible $P^{n_i+1}_{n_r,n_t}$. The second term consists of all excitations,

12 $$\begin{aligned} (n_1,\ldots ,n_i-1,\ldots ,n_r,n_t)\rightarrow (n_1,\ldots ,n_i,\ldots ,n_r,n_t) \end{aligned}$$

and as such carries a positive sign due to the flow of probability *from* $P^{n_i-1}_{n_r,n_t}$ *to* $P_{n_r,n_t}$. This convention is adopted throughout.

$\gamma _i$ is determined by the overlap integral of the incident spectral flux, $f_p(\lambda )$ with dimensions W $\hbox {m}^{-2}$ $\hbox {nm}^{-1}$, with the absorption spectrum of the pigment,

13 $$\begin{aligned} \gamma _i=\int _{0}^{\infty }\frac{\lambda }{hc} f_p\left( \lambda ;T_s,a_{\text {sp}}\right) A_{i}\left( \lambda \right) d\lambda \end{aligned}$$

where the factor $\lambda /hc$ ensures $\gamma _i$ has units of photons $\hbox {m}^{-2}$ $\hbox {s}^{-1}$. We shall assume that the pigment has a Gaussian absorption profile,

14 $$\begin{aligned} A_{i}\left( \lambda ;\sigma _i,\lambda _i^p,w_i\right) =\sigma _i\frac{1}{w_i\sqrt{2\pi }}\text {exp}\left( -\frac{\left( \lambda -\lambda _i^p\right) ^2}{2{w_i}^2}\right) \end{aligned}$$

where $\lambda _i^p$ and $w_i$ are the peak wavelength and standard deviation (hereafter the ’width’) and $\sigma _i$ is the *integrated absorption cross-section*. The absorption spectra of photosynsthetic pigments such as (bacterio)chlorophyll, pheophytin, bilins, etc. tend to be dominated by a Gaussian peak with additional lesser vibronic peaks which we neglect (see Fig. 1E). We also neglect direct photo-excitation of the RC, purely to reduce model complexity. This is justified since the RC typically makes up a very small fraction ($< 1\%$) of the total pigment content of the photosystem.

Excitation dissipation consists of non-photosynthetic de-excitation processes such as fluorescence and non-radiative decay. We combine all of these processes into a single *dissipation rate*, $k_{\text {diss}}$,

15 $$\begin{aligned} \begin{aligned} \left[ \frac{d}{dt}P_{n_r,n_t}(t)\right] _{\text {diss}}&=-k_{\text {diss}}\left( n_r+\sum _{i}n_i\right) P_{n_r,n_t}(t)\\&\quad +k_{\text {diss}}\left( n_r+1\right) P_{n_r+1,n_t}(t)\\ {}&\quad +k_{\text {diss}}\sum _i\left( n_i+1\right) P_{n_r,n_t}^{n_i+1}(t) \end{aligned} \end{aligned}$$

Note that we neglect dissipation of the charge-separated state in the trap (which would occur via non-radiative charge re-combination) as evolution has ensured this very slow (if it were fast it would defeat the point of a photosystem).

The kinetics of excitation trapping and electron output depend explicitly on whether the trap is already occupied (hereafter “closed”),

16 $$\begin{aligned} \left[ \frac{d}{dt}P_{n_r,0}(t)\right] _{\text {trap}}=-n_rk_{\text {trap}}P_{n_r,0}(t) \end{aligned}$$

or unoccupied (hereafter "open"),

17 $$\begin{aligned} \left[ \frac{d}{dt}P_{n_r,1}(t)\right] _{\text {trap}}=-k_{\text {out}}P_{n_r,1}(t)+\left( n_r+1\right) k_{\text {trap}}P_{n_r+1,0}(t) \end{aligned}$$

The timescale of RC trapping occurs on the order of ${k_{\text {trap}}}^{-1}<10$ ps, essentially, with a value of $\sim 5$ ps for PSII (Broess et al. 2006) and $\sim$1–2 ps for PSI (Akhtar et al. 2021). Fast trapping is a universal requirement of antennae which ensures energy conversion out-competes wasteful decay processes. Since we are interested in an oxygenic antennae, we will assume the value of ${k_{\text {trap}}}^{-1}\sim 5$ ps. The timescale for acceptor reduction, ${k_{\text {out}}}^{-1}$ is *much* slower and we choose ${k_{\text {out}}}^{-1} \sim 10$ ms as this is approximately the timescale of the H$_2$O oxidation-quinone reduction cycle of PSII (Oja and Laisk 2000)).

For clarity, when considering energy transfer, it is worth separating transfer between the antenna and the RC and transfer between different LHC sub-units,

18 $$\begin{aligned} \begin{aligned} \left[ \frac{d}{dt}P_{n_r,n_t}(t)\right] _{\text {trans}}=\left[ \frac{d}{dt}P_{n_r,n_t}(t)\right] _{i-r}+\left[ \frac{d}{dt}P_{n_r,n_t}(t)\right] _{i-j} \end{aligned} \end{aligned}$$

The kinetics of antenna-RC transfer are,

19 $$\begin{aligned} \begin{aligned}&\left[ \frac{d}{dt}P_{n_r,n_t}(t)\right] _{i-r}\\ {}&\quad =-\sum _{i}\left( K_{n_i,n_r}^{n_i+1,n_r-1}+K_{n_i,n_r}^{n_i-1,n_r+1}\right) P_{n_r,n_t}(t)\\&\qquad +\sum _{i}\left( K_{n_i+1,n_r-1}^{n_i,n_r}P_{n_r-1,n_t}^{n_i+1}(t)+K_{n_i-1,n_r+1}^{n_i,n_r}P_{n_r+1,n_t}^{n_i-1}(t)\right) \end{aligned} \end{aligned}$$

where the rate constant $K_{n_i,n_r}^{n_i-1,n_r+1}$ refers to the transition,

20 $$\begin{aligned} \left( n_1,n_2,\ldots ,n_i\ldots ,n_r,n_t\right) \rightarrow \left( n_1,n_2,\ldots ,n_i-1\ldots ,n_r+1,n_t\right) \end{aligned}$$

the transfer of an excitation *from* LHC sub-unit *i* *to* the RC. Similarly, the kinetic of energy transfer between LHC sub-units are given by,

21 $$\begin{aligned} \begin{aligned}&\left[ \frac{d}{dt}P_{n_r,n_t}(t)\right] _{i-j}\\ {}&\,\,=-\sum _{i,j}\left( K_{n_i,n_j}^{n_i-1,n_j+1}+K_{n_i,n_j}^{n_i+1,n_j-1}\right) P_{n_r,n_t}(t)\\&\quad\, + \sum _{i,j}\left( K_{n_i+1,n_j-1}^{n_i,n_j}P_{n_r,n_t}^{n_i+1,n_j-1}(t)\right. \\ {}&\quad\, \left. +K_{n_i-1,n_j+1}^{n_i,n_j}P_{n_r,n_t}^{n_i-1,n_j+1}(t)\right) \end{aligned} \end{aligned}$$

Having defined all of the necessary parameters and rate constants (see below) we can numerically solve the equations of motion in the steady state,

22 $$\begin{aligned} \frac{d}{dt}\mathcal {P}_{n_r,n_t}=0 \end{aligned}$$

subject to the constraints,

23 $$\begin{aligned}&\sum _{n_i,n_r,n_t}\mathcal {P}_{n_r,n_t}(t)=1 \end{aligned}$$

24 $$\begin{aligned}&0\le P_{n_r,n_t}(t)\le 1 \end{aligned}$$

where $\mathcal {P}_{n_r,n_t}$ denotes the *steady state* probability. From the set of $\mathcal {P}_{n_r,n_t}$ we can derive the *average occupancies*,

25 $$\begin{aligned} \langle n_t \rangle&=\sum _{n_i,n_r}\mathcal {P}_{n_r,1} \end{aligned}$$

26 $$\begin{aligned} \langle n_r \rangle&=\sum _{n_i,n_r}n_r \left( \mathcal {P}_{n_r,0}+\mathcal {P}_{n_r,1}\right) \end{aligned}$$

27 $$\begin{aligned} \langle n_i\rangle&=\sum _{n_i,n_r}n_i \left( \mathcal {P}_{n_r,0}+\mathcal {P}_{n_r,1}\right) \end{aligned}$$

which in turn are used to derive three parameters related to the overall efficacy of the supercomplex. The first is the *electron output rate*,

28 $$\begin{aligned} \nu _e=k_{\text {out}}\langle n_t\rangle \end{aligned}$$

which gives the steady state flux of electrons out of the system (assuming a limitless supply of acceptor molecules). This can be related to (indirect) measurements of PSII output by, for example, an oxygen electrode. The second parameter is the absolute quantum efficiency of the super-complex

29 $$\begin{aligned} \phi _e=\lim _{\gamma _i\rightarrow 0}\left[ \frac{\nu _e}{\nu _e+k_{\text {diss}}\left( \langle n_r\rangle +\sum _{i}\langle n_i\rangle \right) }\right] \end{aligned}$$

which is the fraction of captured photons that go on to generate an electron in the limit of low (i.e. non-saturating) light. This is not directly comparable to the *PSII quantum efficiency*, $\Phi _{PSII}$, as measured by a Pulse Amplitude Modulated (PAM) chlorophyll fluorometer (Baker and Rosenqvist 2004; Semer et al. 2019), with the latter measuring something closer to the efficiency of excitation trapping. $\phi _e$ therefore represents an underestimate of $\Phi _{PSII}$. Finally we calculate $\nu _e/N_p$, where $N_p$ is the total number of pigments in the antenna, as a crude measure of the ‘biological efficiency’. Maintaining a large array of pigments will come at some metabolic cost to the organism, so the electron output *per pigment* is another useful metric of antenna performance. For PSII in plants a ratio of 300:1 antenna:RC pigments is often quoted (Melis and Anderson 1983). If we assume an electron output rate in the range $\nu _e=1-100$ $\hbox {s}^{-1}$, then for plants $\nu _e/N_p \sim 0.003-0.3$ $\hbox {s}^{-1}$.

#### Energy transfer rate constants

The energy transfer rate constants are the critical parameters in our model and depend on a number of factors,

30 $$\begin{aligned} K_{n_i,n_j}^{n_i-1,n_j+1}=k_{\text {hop}} n_i A_{ij} \phantom {.}\rho \left( E_i-E_j\right) f\left( \Delta F_{n_i,n_j}^{n_i-1,n_j+1}\right) \end{aligned}$$

$k_{\text {hop}}$, is some intrinsic hopping rate that sets the overall timescale. It will depend on the fine molecular detail of the antenna, including size, shape and pigment density of the LHC sub-units, the optical and electronic properties of the pigments, etc. While making no assumption regarding these factors, we can say that an effective antenna would require $k_{\text {hop}}>>k_{\text {diss}}$. As such we set ${k_{\text {hop}}}^{-1}\sim 10$ ps which is also the typical timescale of inter-sub-unit energy transfer in real photosystems (Novoderezhkin et al. 2011). The second term, $A_{ij}$, are the elements of the *adjacency matrix*,

31 $$\begin{aligned} A_{ij}= {\left\{ \begin{array}{ll} 1\text { for } i \text { and } j \text { connected}\\ 0\text { otherwise} \end{array}\right. } \end{aligned}$$

which defines whether two neighbouring sub-units are close enough to exchange energy.

$\rho \left( E_i-E_j\right)$ is the *density of states* that enforces energy conservation on the transfer process. Energy transfer between pigment with different $\lambda _i^p$ is only possible because fluctuations in their excitation energies will periodically bring them into resonance. The size of these fluctuations is reflected in the broadness of their respective absorption lineshapes, $A_i(\lambda )$, and the degree of resonance is approximately captured by the overlap integral,

32 $$\begin{aligned} \rho \left( E_i-E_j\right)&\sim \rho \left( \lambda _i^p,\lambda _j^p\right) \end{aligned}$$

33 $$\begin{aligned}&=\int _{0}^{\infty }d\lambda \tilde{A}_i\left( \lambda ;\lambda _i^p,w_i\right) \tilde{A}_j\left( \lambda ;\lambda _j^p,w_j\right) \end{aligned}$$

where $\tilde{A}_i(\lambda )$ indicates a *normalized* absorption line-shape function. Strictly, it should be the overlap of the *fluorescence* line-shape, $\tilde{F}_i(\lambda )$, of the donor and the absorption line-shape of the acceptor. $\tilde{F}(\lambda )$ is the mirror image of $A_i(\lambda )$ plus a small red-shift (the Stokes shift), but since our line-shapes are Gaussian functions and the Stokes shifts of photosynthetic pigments are relatively small, we neglect this detail.

Finally, $f\left( \Delta F_{n_i,n_j}^{n_i-1,n_j+1}\right)$ characterizes the thermodynamics of energy transfer. In the steady state *forward* and *backward* rate constants are related by the detailed balance condition,

34 $$\begin{aligned} \frac{K_{n_i,n_j}^{n_i-1,n_j+1}}{K_{n_i-1,n_j+1}^{n_i,n_j}}=\left( \frac{n_i}{n_j+1}\right) \text {exp}\left( -\frac{\Delta F_{n_i,n_j}^{n_i-1,n_j+1}}{k_B T}\right) \end{aligned}$$

where $\Delta F_{n_i,n_j}^{n_i-1,n_j+1}$ is the (Helmholtz) free energy change associated with the transfer process,

35 $$\begin{aligned} \Delta F_{n_i,n_j}^{n_i-1,n_j+1}=\Delta H_{n_i,n_j}^{n_i-1,n_j+1}-T\Delta S_{n_i,n_j}^{n_i-1,n_j+1} \end{aligned}$$

The enthalpy term, $\Delta H$, is the difference in internal energy of the system before and after the transfer process,

36 $$\begin{aligned} \begin{aligned} \Delta H_{n_i,n_j}^{n_i-1,n_j+1}&=\left( n_i-1\right) E_{i}+\left( n_j+1\right) E_j-n_i E_i - n_j E_j\\&=E_j-E_i\\&=hc\left( \frac{1}{\lambda _j^p}-\frac{1}{\lambda _i^p}\right) \end{aligned} \end{aligned}$$

where $E_i$ is the excitation energy of the pigments in sub-unit *i* which is inversely proportional to $\lambda _i^p$.

The entropy change, $\Delta S$, can be understood as follows. Consider two coupled LHC sub-units with $N_i$ and $N_j$ pigment states respectively, of which $n_i\le N_i$ and $n_j\le N_j$ are occupied. Assuming the excitations can equally and rapidly sample all of the pigments in their respective LHCs, the combined entropy of the two is,

37 $$\begin{aligned} S_{n_i,n_j}=&k_{B}\text { ln}\left[ W(n_i,N_i)W(n_j,N_j)\right] \ \end{aligned}$$

where $W(n_i,N_i)$ is the multiplicity,

38 $$\begin{aligned} W(n_i,N_i)=\frac{N_i!}{n_i!\left( N_i-n_i\right) !} \end{aligned}$$

If one excitation is transferred from $N_i$ to $N_j$ then the entropy change is,

39 $$\begin{aligned} \Delta S_{n_i,n_j}^{n_i-1,n_j+1}=k_{B}\text { ln}\left( \frac{W(n_i-1,N_i)W(n_j+1,N_j)}{W(n_i,N_i)W(n_j,N_j)}\right) \end{aligned}$$

Implicit in this is the assumption that excitons equilibriate *within* each sub-unit much faster than the typical timescale of energy transfer between then. In Supplementary Fig. S1, we show that this holds so long as roughly $N_{i} < 100$, a pattern generally seen in real LHC proteins (e.g. Zheng et al. 2021; Su et al. 2017).

$\Delta F$ is antisymmetric with respect to reversal of the the transfer process,

40 $$\begin{aligned} \Delta F_{n_i,n_j}^{n_i-1,n_j+1}=-\Delta F^{n_i,n_j}_{n_i-1,n_j+1} \end{aligned}$$

which gives energy transfer a thermodynamically favoured direction. This is encoded into the function,

41 $$\begin{aligned} f\left( \Delta F_{n_i,n_j}^{n_i-1,n_j+1}\right) = {\left\{ \begin{array}{ll} 1\text { for } \Delta F_{n_i,n_j}^{n_i-1,n_j+1}\le 0\\ \\ \text {exp}\left( -\beta \Delta F_{n_i,n_j}^{n_i-1,n_j+1}\right) \text { for } \Delta F_{n_i,n_j}^{n_i-1,n_j+1}>0 \end{array}\right. } \end{aligned}$$

where $\beta =1/k_B T$ is the inverse thermodynamic temperature. In essence, if the forward transfer process, $K_{n_i,n_j}^{n_i-1,n_j+1}$, is thermodynamically favourable ($\Delta F<0$), then the backward rate is unfavourable ($\Delta F>0$) and subject to a multiplicative *Boltzmann penalty*. In Fig. 1F we sketch the balance between enthalpy and entropy. In LHC 1 (green) the exciton can sample 4 pigments, while it can only sample 2 when it transfers to LHC 2 (yellow). This constitutes an unfavourable reduction in entropy, though this can be compensated for providing the transfer is also associated with a significant decrease in enthalpy.

#### The single excitation regime

Since $k_{\text {diss}}$ and $k_{\text {trap}}$ are much faster than $\gamma _i$, even in very bright light, we may assume the *single excitation regime*,

42 $$\begin{aligned}&n_i=0,1 \end{aligned}$$

43 $$\begin{aligned}&n_r=0,1 \end{aligned}$$

44 $$\begin{aligned}&\langle n_r\rangle +\sum _i \langle n_i \rangle \le 1 \end{aligned}$$

In other words, the photosystem can contain a maximum of one excitation at any time, though the trap can be either open or closed. This allows us to neglect multi-excitation terms in our model and if we adopt notation,

45 $$\begin{aligned} P_{0,0,n_t}&=P\left( n_1=0,n_2=0,\ldots ,n_i=0,\right. \nonumber \\&\quad \left. \ldots ,n_{N_\text {LHC}}=0,n_r=0,n_t\right) \end{aligned}$$

46 $$\begin{aligned} P_{1_i,0,n_t}&=P\left( n_1=0,n_2=0,\ldots ,n_i=1,\right. \nonumber \\&\quad \left. \ldots ,n_{N_\text {LHC}}=0,n_r=0,n_t\right) \end{aligned}$$

47 $$\begin{aligned} P_{0,1,n_t}&=P\left( n_1=0,n_2=0,\ldots ,n_i=1,\right. \nonumber \\&\quad \left. \ldots ,n_{N_\text {LHC}}=0,n_r=1,n_t\right) \end{aligned}$$

then our equations of motion reduce to,

48 $$\begin{aligned}&\begin{aligned} \frac{d}{dt}P_{0,0,0}(t)&=-\sum _i \gamma _i N_i(1-n_i)P_{0,0,0}(t)\\&\quad +k_{\text {diss}}\left( P_{0,1,0}(t)+\sum _i P_{1_i,0,0}(t)\right) \\&\quad +k_{\text {out}}P_{0,0,1}(t) \end{aligned} \end{aligned}$$

49 $$\begin{aligned}&\begin{aligned} \frac{d}{dt}P_{0,0,1}(t)&=-\left( \sum _i \gamma _i N_i(1-n_i)+k_{\text {out}}\right) P_{0,0,1}(t) \\ {}&\quad +k_{\text {diss}}\left( P_{0,1,1}(t)+\sum _i P_{1_i,0,1}(t)\right) \\&\quad +k_{\text {trap}}P_{0,1,0}(t) \end{aligned} \end{aligned}$$

50 $$\begin{aligned}&\begin{aligned} \frac{d}{dt}P_{1_i,0,0}(t)&=-\left( k_{\text {diss}}+k_{i\rightarrow r}+\sum _{j}k_{i\rightarrow j}\right) P_{1_i,0,0}(t)\\&\quad +\gamma _iN_i(1-n_i)P_{0,0,0}(t)+k_{r\rightarrow i}P_{0,1,0}(t)\\&\quad +\sum _j k_{j\rightarrow i}P_{1_j,0,0}(t)\\&\quad +k_{\text {out}}P_{1_i,0,1}(t) \end{aligned} \end{aligned}$$

51 $$\begin{aligned}&\begin{aligned} \frac{d}{dt}P_{1_i,0,1}(t)&=-\left( k_{\text {diss}}+k_{\text {out}}+k_{i\rightarrow r}+\sum _{j}k_{i\rightarrow j}\right) P_{1_i,0,1}(t)\\&\quad +\gamma _iN_i(1-n_i)P_{0,0,1}(t)+k_{r\rightarrow i}P_{0,1,1}(t)\\&\quad +\sum _{j}k_{j\rightarrow i}P_{1_j,0,1}(t)\\&\quad +k_{\text {trap}}P_{1_i,1,0}(t) \end{aligned} \end{aligned}$$

52 $$\begin{aligned}&\begin{aligned} \frac{d}{dt}P_{0,1,0}(t)&=-\left( k_{\text {diss}}+k_{\text {trap}}+\sum _i k_{r\rightarrow i}\right) P_{0,1,0}(t)\\&\quad +\sum _{i}k_{i\rightarrow r}P_{1_i,0,0}(t)\\&\quad +k_{\text {out}}P_{0,1,1}(t) \end{aligned} \end{aligned}$$

53 $$\begin{aligned}&\begin{aligned} \frac{d}{dt}P_{0,1,1}(t)&=-\left( k_{\text {diss}}+k_{\text {out}}+\sum _{i}k_{r\rightarrow i}\right) P_{0,1,1}(t)\\&\quad +\sum _{i}k_{i\rightarrow r}P_{1_i,0,1}(t) \end{aligned} \end{aligned}$$

where the rate constants are defined as

54 $$\begin{aligned} k_{i\rightarrow j}\equiv K_{1_i,0_j}^{0_i,1_j} . \end{aligned}$$

The thermodynamic quantities also simplify:

55 $$\begin{aligned}&\Delta H_{1_i,0_j}^{0_i,1_j}\equiv \Delta H_{i\rightarrow j}=hc\left( \frac{1}{\lambda ^p_j}-\frac{1}{\lambda ^p_i}\right) \end{aligned}$$

56 $$\begin{aligned}&\Delta S_{1_i,0_j}^{0_i,1_j}\equiv \Delta S_{i\rightarrow j}=k_B \text { ln}\left( \frac{N_j}{N_i}\right) \end{aligned}$$

### Exploring different light-harvesting structures around different stars

#### An illustrative model: diminishing returns to increasing antenna size

**Fig. 2:**
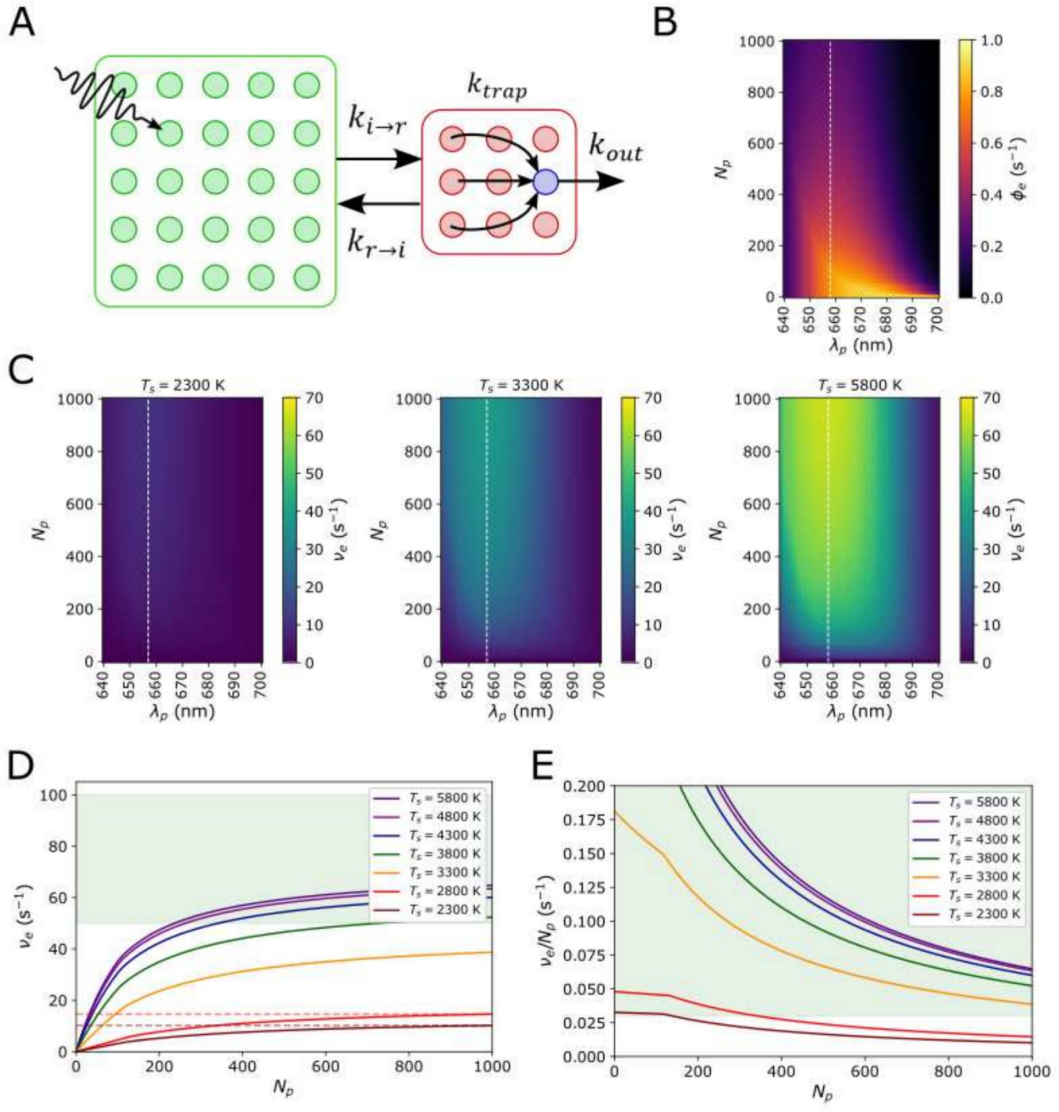
**A** A schematic of a simplified photosystem. An LHC sub-unit (shown in green) containing a variable number, $N_p$, of identical pigments with a variable absorption peak, $\lambda _p$, coupled to an oxygenic RC with absorption peak $\lambda _{p}^{r}=680$ nm (shown in red). Arrows indicate exciton hopping, $k_{i\leftrightarrow r}$; trapping, $k_{\text {trap}}$; and the reduction of the electron carrier, $k_{\text {out}}$. **B** A heatmap of the absolute quantum efficiency of the antenna, $\phi _e(N_p,\lambda _p)$. This is independent of light intensity and therefore identical for all $T_s$. The white dashed line indicates the antenna absorption peak, $\lambda _p^{\text {opt}}\sim 659$ nm, that gives the maximum $\nu _e$. **C** Heatmaps of $\nu _e(N_p,T_s)$ for $T_s=2300$, 3300, and 5800 K. **D** $\nu _e(N_p)$ calculated for $\lambda _p^{\text {opt}}\sim 659$ nm for the full range of $T_s$. The green region indicates the region $\nu _e\ge \nu _e^{\text {max}}/2$ **E** The electron output *per pigment*, $\nu _e/N_p$ as a function of $N_p$ ($\lambda _p^{\text {opt}}\sim 659$ nm) for the full range of $T_s$. The green region indicates $\nu _e/N_p > 0.03$ $\hbox {s}^{-1}$, an approximate lower limit for oxygenic photosynthesis on Earth

We first consider a very simple and purely illustrative supercomplex of a RC coupled to a single LHC of variable size. The RC is assumed to contain $N_r=10$ identical pigments with absorption peak $\lambda _p^r=680$ nm, while the antenna is of variable size, $N_p$, and absorption peak, $\lambda _i^p$ (see Fig. 2A). For several stellar temperatures in the range 2300 K $\le T_s\le 5800$ K we calculate the electron output, $\nu _e(N_i,\lambda _i^p)$, and efficiency, $\phi _e(N_i,\lambda _i^p)$ for varying $N_i$ and $\lambda _i^p$. Here we do not restrict $N_p$ to any upper limit, since we are illustrating that increasing antenna size by adding more and more pigments to LHC initially increases $\nu _e$ but soon runs into steep diminishing returns due to transfer to the RC becoming severely entropy-limited.

#### A ‘modular’ antenna system

**Fig. 3:**
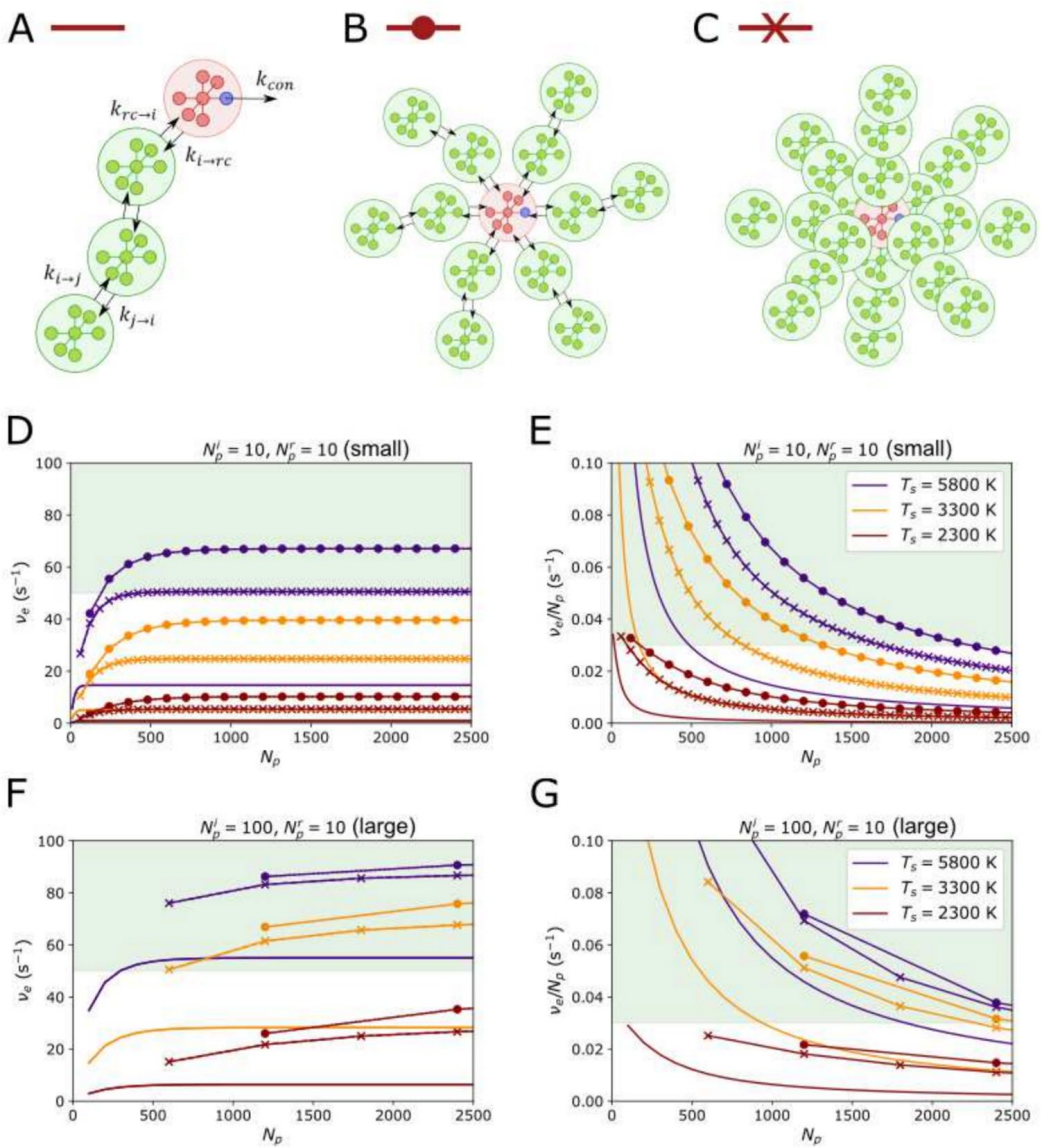
**A** A schematic of a *linear, modular photosystem*. The RC is connected to a single ($N_b=1$) chain of $n_{s}$ identical antenna subunits which each contain $N_p^i$ identical pigments with peak absorbance wavelength, $\lambda _p^i$. **B** A ‘2D’ modular antenna with $N_b=6$ identical branches radiating out from a RC. **C** A ‘3D’ antenna consists of $N_b=12$ identical branches radiating out from the RC. The figure shows only 9 simply for clarity. **D** The electron output rate, $\nu _e$, as a function of *total number of pigments*, $N_p$, for an increasing increasing number of ‘small’ antenna subunits ($N_p^i=N_p^r=10$). We consider a linear (solid line), 2D (line and crosses) and 3D (line and dots) modular photosystem, for $T_s=5800$ (indigo), 3300 (orange), and 2300 K (maroon). Each point represents the addition of 1 antenna sub-unit to *every* branch in the system. The green region indicates the region $\nu _e^{\text {max}}/2 \le \nu _e^{\text {max}}$. **E** The *electron output rate per pigment*, $\nu _e/N_p$, for the same combinations of photosystem structure and $T_s$. The green region indicates $\nu _e/N_p > 0.03$ $\hbox {s}^{-1}$. **F** The same as D. but for ‘large’ antenna sub-units ($N_p^i=100$, $N_p^r=10$). **G** Same as (**E**). but for large antenna sub-units

We then consider a more realistic antenna composed of a set of identical LHC sub-units (hence ‘modular’) of fixed size. We again assume an RC with $N_r=10$ pigment states and $\lambda _p^r=680$ nm, and consider ‘small’ and ‘large’ LHC sub-units which contain $N_i=10$ and 100 pigment states respectively. Its important to remember that, in addition to explicit inclusion in the definition of $k_{i\rightarrow j}$, entropy will have an implicit influence on the stochastic diffusion of the exciton across the entire antenna. As such we consider a *branched* structure, in which chains of LHCs radiate out from a central RC. Of course, other antenna structures are possible, such as a cubic or hexagonal lattices, but these will be less effective since the exciton would have greater freedom to ‘wander about’ before hitting the RC. We consider photosystems that have $N_b=1$, 6 and 12 branches (see Fig. 3A–C). We can label $N_b=1$ a 1-dimensional (1D) antenna. If we assume that the branches are close-packed around the RC then $N_b=6$ can be thought of as planar or 2D, and by the same logic $N_b=12$ can be loosely considered 3D.

As before we vary the antenna absorption peak, $\lambda _i^p$, and antenna size, with the latter achieved by adding LHC sub-units to the ends of the branches. We calculate $\nu _e(N_p,\lambda _{p}^i)$ and $\phi _e(N_p,\lambda _{p}^{i})$ for several values of $T_s$, where $N_p=\sum _{i}N_i$.

#### A funnel antenna system

**Fig. 4:**
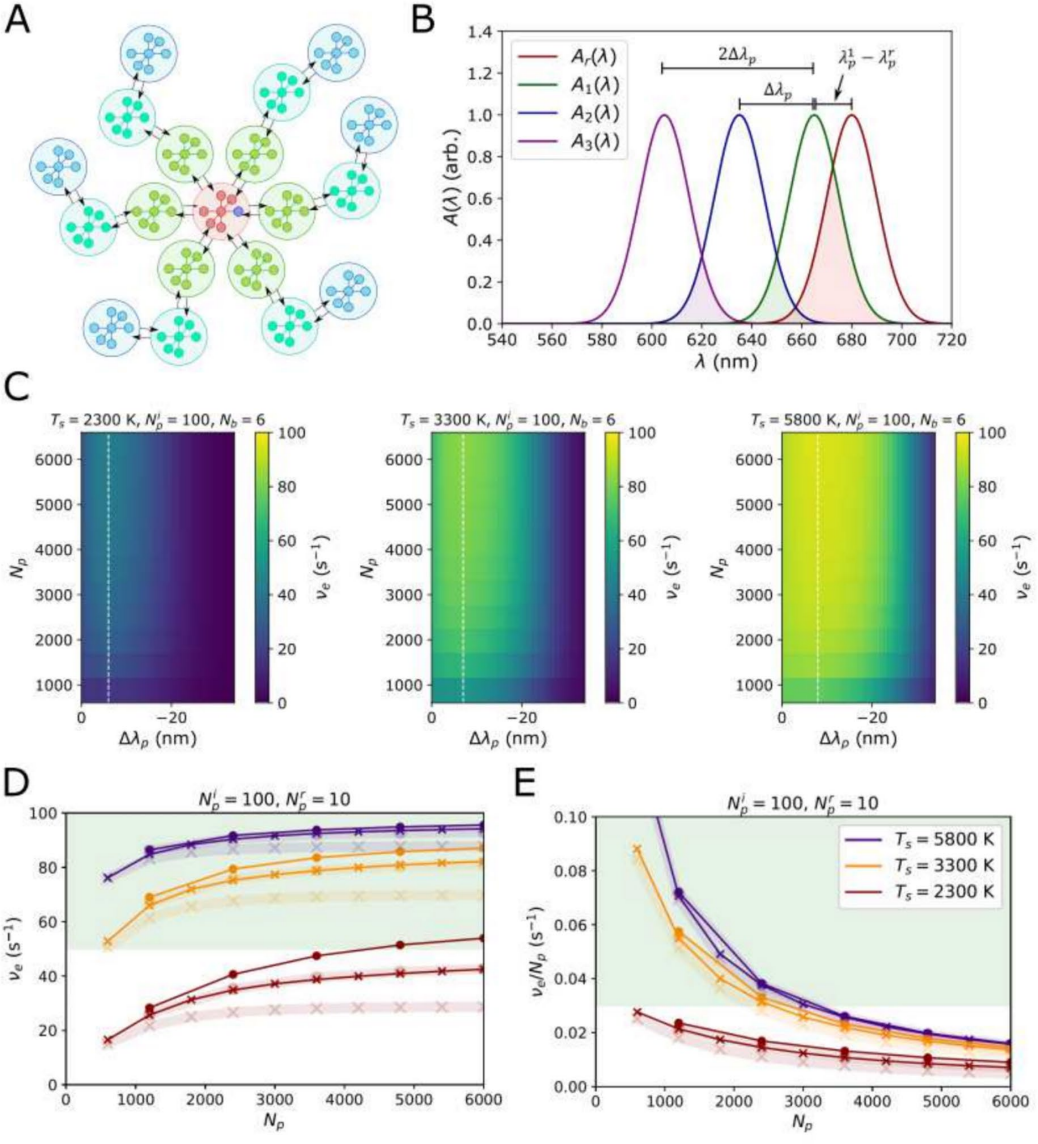
**A** Schematic of a funnel antenna in which different sub-units bind different pigment types and the arrangement of these different sub-units is highly conserved. The different colors signify that bluer pigments are bound further out from the RC. **B** An illustrative plot of the absorption profiles of a chain of 3 progressively shifted antenna sub-units connected to the RC. **C** Heat-maps of $\nu _e\left( \Delta \lambda _p,N_p\right)$ for $T_s=2800$, 3300, and 5800 K, where $N_b=6$ and $N_p^i=10N_p^r$. Not shown are the same data for $N_b=12$ which shows exactly the same trend but a slightly higher maximum $\nu _e$. The white dashed line indicates the most optimum $\Delta \lambda _p$. **D** Plots of $\nu _e(N_p)$ for optimum $\Delta \lambda _p$. The labelling is the same as Fig. 3. The fade curves are for a modular antenna, repeated here for comparison. The green region indicates the region $\nu _e^{\text {max}}/2 \le n_e^{\text {max}}$ **E** $\nu _e/N_p$ for the same structured antenna configurations. The green region indicates $\nu _e/N_p > 0.03$ $\hbox {s}^{-1}$

Lastly, we attempt to negate the diminishing returns of an increasingly large antenna by introducing a variable enthalpy gradient within the antenna. For the same antenna structures as for the modular antenna ($N_b=1$, 6, and 12) we assume the inner-most LHCs have absorption peak $\lambda _1^p$. Moving out to the end of the branches we then add a progressive blue-shift, $\Delta \lambda _p$ so that,

57 $$\begin{aligned} \lambda _1^p&=\lambda \end{aligned}$$

58 $$\begin{aligned} \lambda _2^p&=\lambda -\Delta \lambda _p \end{aligned}$$

59 $$\begin{aligned} \lambda _3^p&=\lambda -2\Delta \lambda _p \end{aligned}$$

and so on. This is represented as a sketch in Fig. 4A and in terms of absorption spectra in Fig. 4B.

## Results

### Orbital distances and incident spectral fluxes

**Table 1:** The maximum, $a_{\text {sp}}^{\text {max}}\left( T_{p}=273 \text { K}\right)$, minimum, $a_{\text {sp}}^{\text {min}}\left( T_{p}=373 \text { K}\right)$, and midpoint, $a_{\text {sp}}^{\text {mid}}$, habitable distances for stellar models defined by stellar temperature, $T_{s}$, and radius, $R_{s}$

$T_{s}$ (K)	$R_{s} \left( R_{\odot }\right)$	$a_{\text {sp}}^{\text {max}}$ (a.u.)	$a_{\text {sp}}^{\text {min}}$ (a.u.)	$a_{\text {sp}}^{\text {mid}}$ (a.u.)
2300	0.117	0.019	0.010	0.015
2600	0.133	0.028	0.0.015	0.021
2800	0.254	0.062	0.033	0.048
3300	0.299	0.102	0.055	0.078
3800	0.613	0.276	0.148	0.212
4300	0.694	0.400	0.214	0.307
4800	0.775	0.557	0.299	0.428
5800	0.936	0.982	0.526	0.754

Orbital distances are expressed in astronomical units, $1 \text {a.u.}\approx 1.50\times 10^{11}$ m, and stellar radii are expressed in terms of the Solar radius, $1 R_{\odot }\approx 6.96\times 10^{8}$ m

The habitable orbital distances, $a_{\text {sp}}^{\text {min}}\le a_{\text {sp}}\le a_{\text {sp}}^{\text {max}}$, derived using Eq. (2) are listed in Table 1. Note that Earth’s orbit ($1 \text {a.u.} \sim 14.96\times 10^{10}$ m) lies slightly outside the predicted habitable zone for a $T_{s}=5800$ K star due to neglect of any greenhouse effect. However, these distances are reasonable for qualitative comparison across a range of $T_{s}$. For the mid-point in this range we calculate $f_{p}\left( \lambda ;T_{s}a_{\text {sp}}^{\text {mid}}\right)$ which are plotted in Fig. 1A. In the range $4300 \text { K} \le T_{s} \le 5800 \text { K}$ the irradiance peak is in the PAR region, moving to the typical anoxygenic region ($750 \text { nm}< \lambda < 1000 \text { nm}$) for $3300 \text { K} \le T_{s} \le 3800 \text { K}$, and then red-shifting out of the window for photo-autotrophy on Earth for $T_{s}\le 2800$ K. However, it should be noted that there is non-negligible irradiance in the PAR region for all $T_{s}$.

### The illustrative model

Figure 2B, C show 2D plots of $\phi _e(\lambda _p,N _p)$ and $\nu _e(\lambda _p,N_p)$ respectively. $\phi _e(\lambda _p,N_p)$ is an intrinsic property of the antenna and is thus independent of $f_p(\lambda ;T_s)$. $\nu _e(\lambda _p,N_p;T_s)$, however, is dependent on the type of star, and while we only show $T_s=2800$, 3300, and 5800 K it is trivial to interpolate between these values. $\phi _e$ falls off steeply with increasing $N_p$ as the entropic penalty of transferring energy from a huge antenna to a small RC becomes prohibitive. This can be somewhat mitigated by blue-shifting the antenna pigments. However, this soon runs into the problem of the spectral overlap, $\rho \left( \lambda _p,\lambda _p^r\right)$, decreasing so much as to make energy transfer slower than excitation decay, $k_{i\rightarrow r}<k_{\text {diss}}$. If we look at Fig. 2C we see that for all $T_s$ the maximum $\nu _e(\lambda _p,N_p)$ is achieved when $\lambda _p\sim 659$ nm, compared to the RC absorption peak of $\lambda _p^r\sim 680$ nm. This represents the optimum balance between ensuring a favourable free energy decrease from the antenna to the RC, while maintaining some degree of spectral overlap. We note that shifting the antenna absorption peak into the far-red ($\lambda _p>680$ nm) does not improve the performance of the antenna in M-dwarf light, despite an abundance of photon flux.

Figure 2D, E plot $\nu _e$ and $\nu _e/N_p$ respectively for the optimum $\lambda _p=659\text { nm}$. The green shaded area in Fig. 2D marks where $\nu _e$ exceeds half the maximum value of $\nu _e^\text {max}=100$ $\hbox {s}^{-1}$, while in Fig. 2E is denotes $0.03<\nu _e/N_p<0.03$ $\hbox {s}^{-1}$. There is a sharp change over from one type of behaviour to another at $T_s\sim 3300-3800$ K, which represent the hotter M-dwarfs. For the coolest M-dwarfs ($T_s\le 2800$ K) the antenna *cannot be significantly improved* by simply increasing it’s size, even with the optimal enthalpy drop between antenna and RC. An antenna consisting of $N_p=1000$ pigments is needed to achieve the same output that is possible with $N_p<20$ for hotter stars, and this comes at the expense of a very small electron-per-pigment rate ($\nu _e/N_p<<0.03$ $\hbox {s}^{-1}$). This is entirely the result of energy transfer from the antenna to the RC becoming entropy-limited. For hotter K and G-type stars ($T_s\ge 4300$ K) this also occurs but there is also a contribution from the the RCs becoming saturated. At $\nu _e=50$ $\hbox {s}^{-1}$ the RC is closed 50% of the time and it becomes difficult to increase $\nu _e$ further.

Lastly, we note that this type of antenna does not perform particularly well under any type of light. This is simply because the overall structure is poorly ‘designed’, with a narrow bottleneck between the antenna and the RC (Fig. 2A).

### A ‘modular’ antenna

For all three antenna geometries shown in Fig.3A–C we found that, independent of of $T_s$, the optimal absorption peak for the antenna is $\lambda _p^i\sim 665$ nm, slightly redder than for the simplified model. Fig. 3D, E shows $\nu _e$ and $\nu _e/N_p$ respectively for $\lambda _p^i=665$ nm, assuming ‘small’ LHC sub-units ($N_r=N_p=10$). The solid lines correspond to the 1D antenna ($N_b=1$), the lines-plus-crosses to 2D ($N_b=6$), and lines-plus-circles to 3D ($N_b=12$). $N_p$ is incremented by adding one LHC sub-unit to the end of every branch, hence the sparser data-points for the 3D antenna. For clarity, we only show $T_s=2800$, 3300, and 5800 K. The 1D antenna performs poorly even for $T_s=5800$ K, reaching a maximum electron output of $\nu _e<20$ $\hbox {s}^{-1}$, which is even worse than the illustrative model. The latter (unrealistically) assumed instantaneous equilibration over one very large LHC, while in this model the excitation has to hop along a chain of several LHCs before reaching the antenna. Unsuprsingly, if these LHCs are instead arranged into multiple branches, with shorter chains of sub-units and multiple connections points between RC and antenna, then $\nu _e$ increases significantly. However, antenna performance is still very poor ($\nu _e<10$ $\hbox {s}^{-1}$) for $T_s=2300$ K and modest ($\nu _e<50$ $\hbox {s}^{-1}$) for $T_s=3300$ K. The is again due to transfer to the RC becoming entropy-limited. This entropy effect is implicit in the overall structure of the antenna, rather then arising from any explicit entropy penalties applied to the various rates (all sub-units including the antenna are the same size, $N_i=N_j=N_r=10$, and energy).

In Fig. 3F, G we plot $\nu _e(N_p)$ and $\nu _e/N_p$ for ‘large’ LHC sub-units ($N_r=10$, $N_i=100$). For all $T_s$ and $N_b$, having larger but fewer sub-units outperforms the previous structure. For $T_s=5800$ K, values of $\nu _e$ close to the maximum are achievable, with RC saturation being the reason for not hitting $\nu _e=100$ $\hbox {s}^{-1}$ exactly. We see this in the fact that a 3D antenna confers almost no advantage over a 2D one. The electrons-per-pigment rate also fall well within the range we estimated for plant PSII. This improvement is interesting since having $N_1>>N_r$ adds an explicit entropy penalty in addition to the implicit one. Clearly, the reduction in the number of inter-sub-unit hops over-compensates for this. There is also significant improvement for the hotter M-dwarf light ($T_s=3300$ K), with $\nu _e\sim 70$ $\hbox {s}^{-1}$ achievable for a relatively small number of LHC sub-units. For the coolest M-dwarf ($T_s=2300$ K), though somewhat improved, the electron output, $\nu _e<40$ $\hbox {s}^{-1}$, and the electrons-per-pigment, $\nu _e/N_p<0.03$ $\hbox {s}^{-1}$, rates are still rather small.

### A ‘funnel’ antenna

For the 1D, 2D and 3D structure considered for the modular antenna, we assume that $\lambda _p^{1}=665$ nm for the LHC sub-unit closest to the RC (since this was optimum for the previous model). Moving out from the RC we add the progressive blue-shift, which is sketched in Fig. 4A and shown in terms of absorption profiles in Fig. 4B. In Fig. 4C we plot heatmaps of $\nu _e(\Delta \lambda _p,N_p)$, showing only $T_s=2300$, 3300 and 5800 K for $N_b=6$ for clarity. Since they were shown to be the better strategy in the previous model, we consider only ’large’ LHC sub-units ($N_i=100$, $N_r=10$). In all cases the optimal $\Delta \lambda _p\sim 7$ nm, though the distribution is quite broad. Figures 4D, E plot $\nu _e$ and $\nu _e/N_p$ for this optimal shift with the traces from the previous model (modular antenna, Fig. 3) shown as faded line for comparison.

For $T_s=5800$ K a funnel antenna confers almost no advantage over the previous modular one. The PAR flux for a Sun-like star is sufficient to saturate the RC even in with a small, modular antenna. For $T_s=3300$ K the improvement gained from having a funnel antenna rather than a modular one is significant, to the point where the RC saturates. A similar degree of improvement is seen for $T_s=2300$ K, though the maximum achievable electron output rate is only $\nu _e\sim 50$ $\hbox {s}^{-1}$ and requires a very large antenna.

## Discussion

The key conclusions of this work are: (1) For a rocky planet orbiting in the habitable zone of hot stars like our G-type Sun or K-type stars ($T_{s} > 3800$ K), the PAR flux is sufficient to provide maximal electron output, even with a small, modular antenna. No real ‘funnel’ is required other than a small enthalpy drop between the terminal LHC and the RC. (2) At the hottest end of the M-dwarf stars ($T_{s} \sim 3300$ K) it is possible to capture enough light to saturate the RC but a very large funnel-like antenna is required. The electrons-per-pigment rate may be much smaller than for hotter stars, possibly making oxygenic photosynthesis a more ‘expensive’ investment. (3) For the coolest M-dwarf stars ($T_{s} \sim 2300$ K) a moderate electron output rate is feasible, though it struggles to reach the levels possible around hotter stars and requires a very large funnel antenna. (4) In not one of the cases we considered, was electron output rate improved by red-shifting the antenna absorption to longer wavelengths than the RC peak, despite the huge amount of flux available in the NIR region for M-dwarf stars. (5) These conclusions emerge from fundamental thermodynamic constraints on the process of light-harvesting. Rather than an ‘anything is possible’ approach to hypothetical astrobiology, one can place definite limits on the type of processes that are feasible and how they might be realized.

**Fig. 5:**
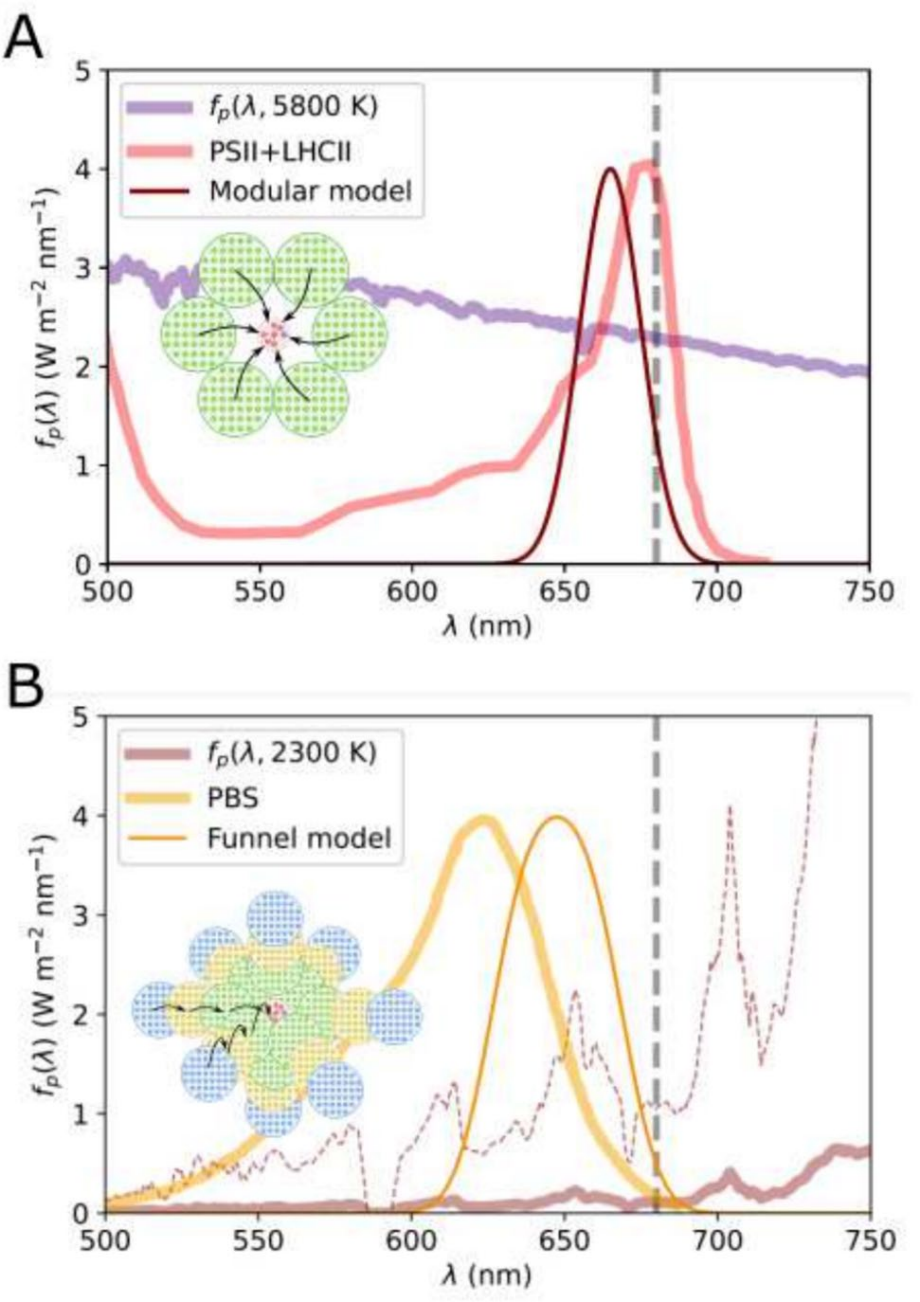
**A** The absorption spectrum (dark red line) of an antenna composed of a ring of 6 identical LHC sub-units, with an absorption maximum at $\lambda _p= 665$ nm, compared to the $Q_y-Q_x$ absorption band of PSII in plants (light red line, digitized from Laisk et al. 2014). Also shown is he spectral flux of $f_p(\lambda , 5800 \text { K})$ (purple line) and the vertical dashed line indicates the position ($\lambda _p^r = 680$ nm) of the RC. Inset is a sketch of this antenna system. **B** The combined absorption spectrum (sharp orange line) of a funnel antenna with 12 branches each of 5 sub-units, and a progressive blue-shift of $\Delta \lambda _p= 8$ nm. This is shown alongside the absorption spectrum of the phycobilisome (PBS) antenna from cyanobacteria *Synechocystis* sp. PCC 6803 (thick orange line, digitized from Lea-Smith et al. 2014). Also shown is the spectral flux of $f_p(\lambda ,2300\text { K})$ in absolute scale (solid red line) and stretched for clarity (dashed red line). Inset is a very rough sketch of this 3D antenna system surrounding the RC

That the irradiant flux from a G-star can support abundant oxygenic photosynthesis is not surprising, given that we already have a real example of this on Earth. The fact this is likely also true for the smaller, cooler, and very orange, K-type stars is more interesting and broadens our search criteria beyond Earth-like planets orbiting Sun-like stars. In both cases our model predicts a *plant-like* antenna: small, modular and with a narrow absorption profile very similar to the red-band of the PSII+LHCII absorption spectrum of higher plants (see Fig. 5A). The antenna peak, $\lambda _p\sim 665$ nm, is selected because it simultaneously provides the free energy difference and a spectral overlap of the antenna and RC, rather than being finely tuned to some local maximum in the irradiant flux. A small modular antenna has the advantage of needing less pigments, taking up less space in the cell, and being inherently adaptable. It is easier to reorganize an antenna system if it is composed of identical sub-units, and under G and K-type light our hypothetical organisms may need to employ such mechanisms to avoid photo-damage from excessive light (Bassi and Dall’Osto 2021; Ruban 2016; Vialet-Chabrand et al. 2017; Li et al. 2021). In terms of detectability of oxygenic photosynthesis, G and K type stars are promising. If photoautotrophs can harvest abundant light with a relatively ‘cheap’ antenna system, then the likelihood of widespread surface populations increases, along with the likelihood of a detectable reflectance edge.

The cooler M-dwarf stars appear to offer a much more limited environment. Even when directly exposed, a very large, funnel antenna is needed to achieve a reasonable electron output, with smaller, modular antennae performing very poorly. This offers an explanation as to why cyanobacteria grow comfortably under simulated M-dwarf light while plants struggle (Battistuzzi et al. 2020, 2023a, b). Our predicted funnel antenna is qualitatively similar to the cyanobacterial PBS, with clusters of progressively blue-shifted pigments radiating out from the RC. The absorption spectrum is also roughly similar, being broader and blue-shifted with respect to PSII+LHCII, though not as blue-shifted as the actual PBS (see Fig. 5B). This makes sense, since the PBS evolved to function in light that is both limiting and blue-shifted (with respect to the sea level flux) due to red-attenuation by water. From Fig. 5B we see that the model funnel antenna overlaps with a local flux maximum at $\sim 650$ nm, though it is significantly broader and also covers a local minimum at $\sim 625$ nm. A modular antenna with the same number of pigments, all narrowly tuned to this local flux maximum performs significantly worse, showing that an enthalpy funnel is beneficial even if it reduces the amount of light absorbed. Overcoming the entropy penalty inherent in a large antenna is critical. In terms of detectable life, this potentially poses problems. A large funnel antenna requires not only lots of pigments but several different *types* of pigment which may need different biosynthetic pathways. It requires more protein to contain/bind these pigment and will presumably take up more space within the cell. This may mean that there is not a lot of *metabolic surplus* to support large amounts of non-photosynthetic tissue, which might limit oxygenic photosynthesis to small organisms and/or marine environments. This in turn could mask any reflectance edges, though being oxygenic they could still affect the atmosphere in a very obvious way. Given the difficulty in harvesting PAR flux and the abundance of NIR light from such stars, it may be anoxygenic photosynthesis is the better evolutionary strategy (Duffy et al. 2023). This would present a different set of potential biosignatures, including reflectance edges in the NIR (Lehmer et al. 2021), but would likely require a planet with an abundance of alternative electron donors to water (e.g. hydrogen sulfide (H$_{2}$S), hydrogen (H$_{2}$), or iron (II) (Fe(II))).

Warmer M-dwarf stars ($T_s\ge 3000$ K) seem to represent a transition between the ‘plant-like’ and ‘cyanobacteria-like’ regimes we have just identified. Interestingly, the transition seems to be rather sharp in terms of stellar temperature, and we could (cautiously) suggest that $T_s > 3000$ K is a reasonable rule-of-thumb for judging whether a particular star is a strong (or less weak) target for a bio-signature survey.

Finally, we note that our model fluxes, $f_p\left( \lambda ;T_s\right)$, strictly represent the light hitting the upper atmosphere of the planet. This flux will be further attenuated by atmospheric absorption/scattering before it hits the planet surface. Although not an exhaustive survey, we explored the effects of atmospheric attenuation on our conclusions in the Supplementary Material. Using the NASA planetary spectrum generator (REFS) (Villanueva et al. 2018) we generated transmission functions for an Earth atmosphere with varying amounts of methane and water. These were chosen since they can both significantly attenuate flux in the PAR region. We found that neither methane (Fig. S2) nor water (Fig. S3), even at very high concentrations, produced any qualitative reduction in $\nu _e$ nor any alteration to the general thermodynamic trends presented in the main article. Of course, shading from clouds, geographical features, and even other organisms would produce highly-modified *local* fluxes. Generally, this won’t *increase* the local surface flux relative to the top-of-atmosphere, and so we can consider out results as upper limits. Planets orbiting G and K-type stars will have a range of light-environments from full sun to heavily shaded most likely selecting for a range of light-harvesting strategies as on Earth. Planets orbiting M-dwarf stars, however, will be much more limited, with even the brightest niches requiring the kind of light-harvesting strategies found in the shade on Earth.

## Supplementary Information

Below is the link to the electronic supplementary material.

Supplementary file 1 (pdf 549 KB)

## Data Availability

The core model code, spectral fluxes, atmospheric transmission spectra, and sundry scripts used to generate graphs are available from https://github.com/QMUL-DuffyLab/Thermal-Antenna-Model (will be made public upon publication).
