# Supplementary material for: Optimizing photosynthetic light-harvesting under stars: simple and general antenna models
Source: Photosynth Res. 2024 Sep 10;162(1):75–92. doi: 10.1007/s11120-024-01118-1 (PMC11413096; doi:10.1007/s11120-024-01118-1)
Supplement: Supplementary file 1 — (pdf 549 KB) [file 11120_2024_1118_MOESM1_ESM.pdf]

Optimizing photosynthetic light-harvesting under stars: Generalized thermodynamic models

Samir Chitnavi[1,2], Callum Gray[1,2], Ifigenia Rousouli[3], Edward Gillen[3], Conrad W. Mullineaux[1], Thomas J. Haworth[3], and Christopher D. P. Duffy[1,2,*]

[1]School of Biological and Behavioural Sciences, Queen Mary University of London, Mile End, London E1 4NS, UK

[2]Digital Environment Research Institute, Queen Mary University of London, Empire House, Whitechapel, London E1 1HH, UK

[3]Astronomy Unit, Queen Mary University of London, Mile End Road, London E1 4NS, UK

*c.duffy@qmul.ac.uk

SUPPLEMENTARY MATERIAL

1. Maximum size of an LHC sub-unit

We consider an LHC sub-unit that is a cubic lattice of N_p identical pigments with arbitrary dimensions,

$$N_p = N_x N_y N_z \quad \text{(S1)}$$

with the pigments indexed $0 \leq i \leq N_p$. Each pigment is associated with an average excitation population, $0 \leq \langle n_i \rangle \leq 1$, and we assume a random pigment, j, is excited at $t = 0$,

$$\langle n_i(0) \rangle = \delta_{ij} \quad \text{(S2)}$$

where δ_{ij} is the *Kronecker delta*. The populations evolve according to,

$$\frac{d}{dt}\langle n_i(t) \rangle = -\sum_{j \neq i} \left(k_{i \to j} \langle n_i(t) \rangle - k_{j \to i} \langle n_j(t) \rangle \right) \quad \text{(S3)}$$

where we assume that the inter-pigment energy transfer rate is $k_{i \to j}{}^{-1} \sim 1$ ps. The system will evolve until the populations reach their equilibrium values,

$$\langle n_i(\tau_{eq}) \rangle = \frac{\exp(-\beta E_i)}{\sum_j \exp(-\beta E_j)}$$

$$= \frac{1}{N_p} \quad \text{(S4)}$$

where the final value of $1/N_p$ arises from the fact that all pigments are identical (same energy, E_i), and τ_{eq} is a characteristic *equilibration time.* Lastly, we can define the rms deviation of the populations from their equilibrium values,

$$\Delta n(t) = \sqrt{\frac{1}{N_p} \sum_i \left(\langle n_i(t) \rangle - \frac{1}{N_p} \right)^2} \quad \text{(S5)}$$

For a large sample of random dimensions, (N_x, N_y, N_z), and initially excited pigments we compute τ_{eq} as the time needed to for the rms deviation to fall to $\Delta n(\tau_{eq}) = 0.01$ (the deviation from equilibrium is no greater than 1%). The distribution of these samples are shown in Fig. S1. We see

that τ_{eq} can vary hugely for a given N_p, due to variations in the shape of the subunit and the initial location of the excitation. The shorter values of τ_{eq} for a given N_p tend to occur when the sub-units is approximately a cube and the excitation starts near the centre. Despite the variations, there is a clear minimum τ_{eq} for a given N_p. We see that for $N_p > 100$ τ_{eq} *must* exceed the inter-sub-unit transfer time of ${k_{hop}}^{-1} \sim 10$ ps. We therefore limit our LHC sub-units size to a maximum of $N_p = 100$.

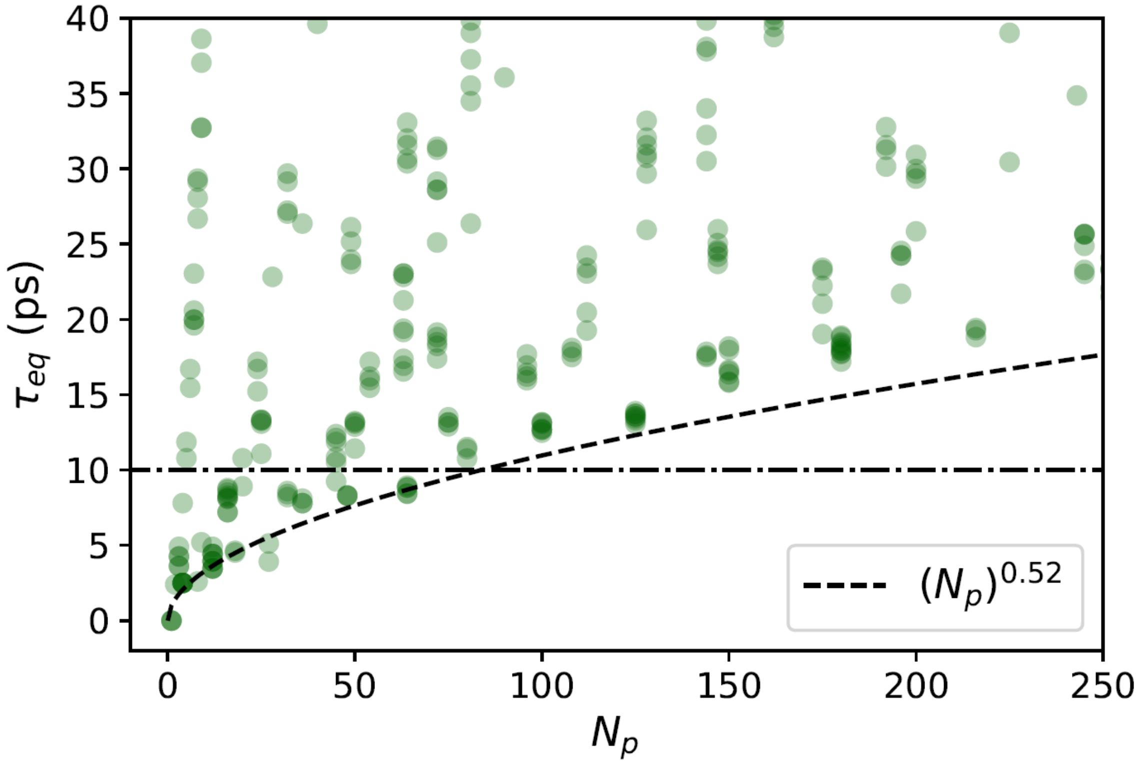

Figure S1: Samples of τ_{eq} such that $\Delta n(\tau_{eq}) = 0.01$, calculated for ramdom antenna dimensions, (N_x, N_y, N_z), and initially excited pigments, $\langle n_i(0) \rangle$. The horizontal dot-and-dash line indicates where $\tau_{eq} \geq 10$ ps, the assumed inter-sub-unit transfer time for our antenna model. The curved dashed line is $f(N_p) = {N_p}^{0.52}$, a visual fit to the minimum τ_{eq} for a given N_p. The two dased lines intersect at $N_p = 83$ though the errors on the visual fit, $f(N_p)$, are quite large.

2. The effect of atmospheric attenuation on photosynthetic performance

We consider the affect of atmospheric attenuation on the antenna performances considered in the main article. Specifically, we consider the presence of variable amounts of water and methane, as these two compounds significantly attenuate the spectral flux in the 400 nm $< \lambda < 700$ nm range relevant to oxygenic photosynthesis.

The atmospheric transmission functions are calculated using the NASA planetary spectrum generator (PSG, Villanueva et al. 2018, 2022). This combines atmospheric models, microphysics databases, radiative transfer, and instrument models for a wide array of applications in the study of radiation transport through planetary atmospheres. We utilised the online version of the tool (https://psg.gsfc.nasa.gov/) to calculate the transmission function through various atmospheres. This simply runs from 1 (complete transmission) through to zero (no transmission) and so is included simply by multiplying the spectrum at the top of the atmosphere by the transmission function.

We consider an Earth-like planet and atmosphere and adjust the abundance of the key absorbers: water and methane. The fiducial abundances are given in table 2. When we modify the Earth atmospheric composition to scale the methane abundance, we offset the change by scaling the nitrogen abundance. When scaling the water abundance we offset it by changing both the nitrogen and oxygen abundance, as summarised in Table 3. For all atmospheric compositions, the abundance of gases is assumed to be the same across all altitudes. We calculate the transmission for the star at zenith, that is along the shortest path through the atmosphere.

Fig. S2 **A.** shows the $f_p(\lambda; T_s)$ for $T_s = 5800$ K, 3300 K, and 2300 K, comparing the unfiltered PHOENIX models of the main article with to fluxes subject to attenuation by an Earth-like atmosphere with 1% and 10% methane. Methane results in strong absorption features at approximately 545, 620, and 670 nm. In absolute terms the attenuation is less apparent at low temperatures. Fig. S2 **B.** shows the performance of a 'modular' antenna (12 branches, large LHC sub-units) in these attenuated fluxes. For $T_s = 5800$ K there is barely any reduction in electron output, since at photon fluxes ν_e is limited by the capacity of the RC rather than the incident flux. For $T_s = 2300$ K an already limited ν_e^{max} is reduced from ~ 40 s^{-1} to ~ 35 s^{-1}. In Fig. S2 **C.** we consider the performance of a 'structured antenna' (12 branches) in the same attenuated fluxes. The effect on ν_e^{max} are similarly quantitative rather than qualitative.

Fig. S3 is the same as Fig. S2, but with 1% and 4% Water instead of the elevated Methane levels. Fig. S3 **A.** shows that water produces quite broad absorption bands at 600 nm, 650 nm and 700 nm. As with Methane though, the attenuation is not sufficient to qualitatively affect photosynthetic performance. That said, this is not an exhaustive survey.

Table 1: Changes in the composition of a Modern Earth-like at atmosphere upon variations in water content. Data taken from the NOAA website (https://www.noaa.gov/jetstream/atmosphere).

$\boldsymbol{H_2O}$	$\boldsymbol{N_2}$	$\boldsymbol{O_2}$
0%	78.084%	20.946%
1%	77.3%	20.7%
2%	76.52%	20.53%
3%	75.74%	20.32%
4%	74.96%	20.11%

Table 2: The assumed atmospheric compositions of a Modern Earth-like atmosphere. Data taken from the NOAA website (https://www.noaa.gov/jetstream/atmosphere).

**Species**	**Abundance**
CO_2	0.0407%
O_2	20.946%
O_3	0.00006%
H_2O	0.8%
CH_4	0.00018%
CO	0.106 ppmv
N_2O	0.305 ppmv
N_2	78.084%

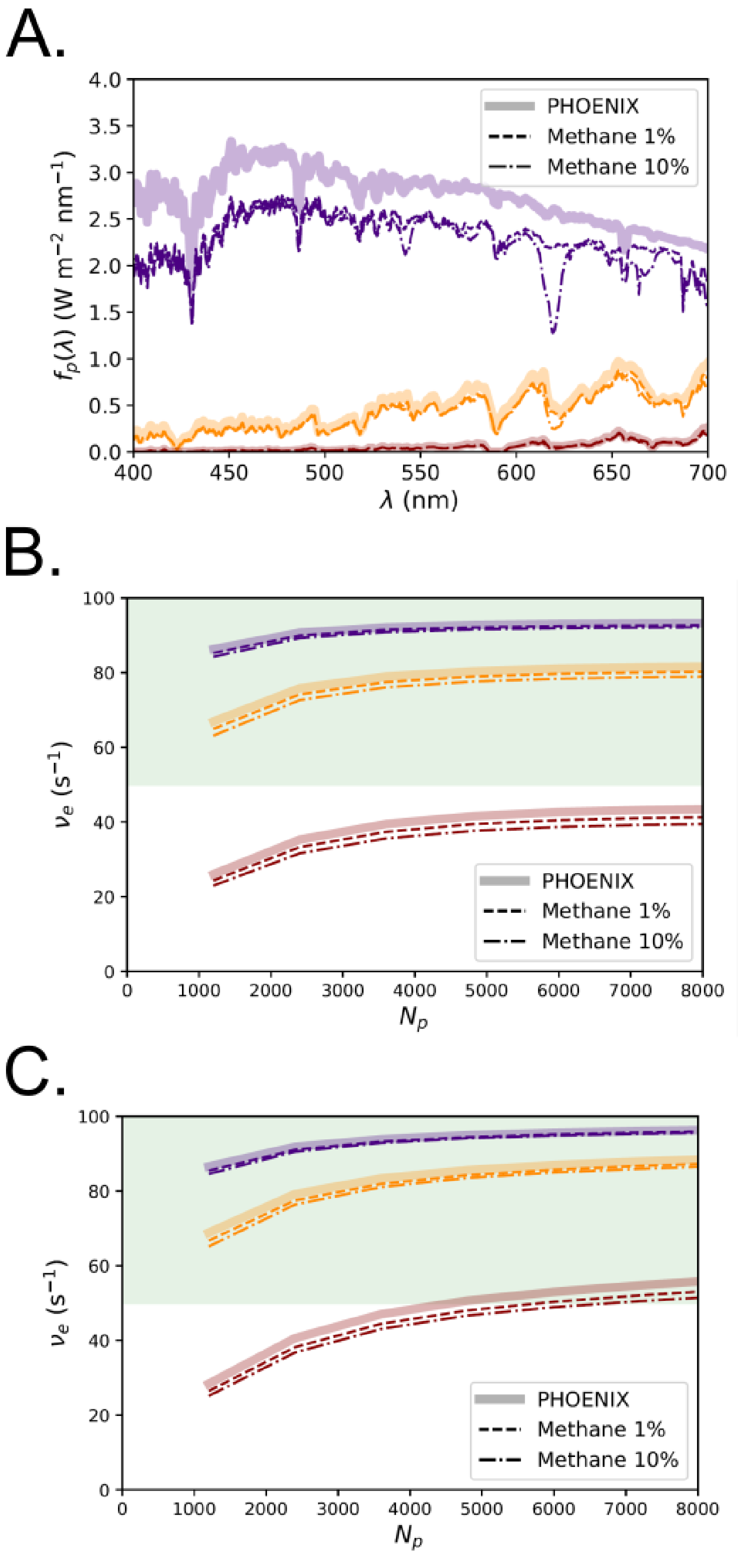

Figure S2: A. The spectral flux for $T_s = 5800$ K (indigo), 3300 K (orange), and 2300 K (dark red), for the unfiltered PHOENIX model (thick, faded line), transmitted through an Earth-like atmosphere with 1% Methane (dashed line) and 10% Methane. **B.** The electron output for a 'modular' antenna with large LHC sub-units ($N_p^i = 100$) and 12 branches. **C.** The electron output for a 'structured' antenna with large LHC sub-units ($N_p^i = 100$) and 12 branches.

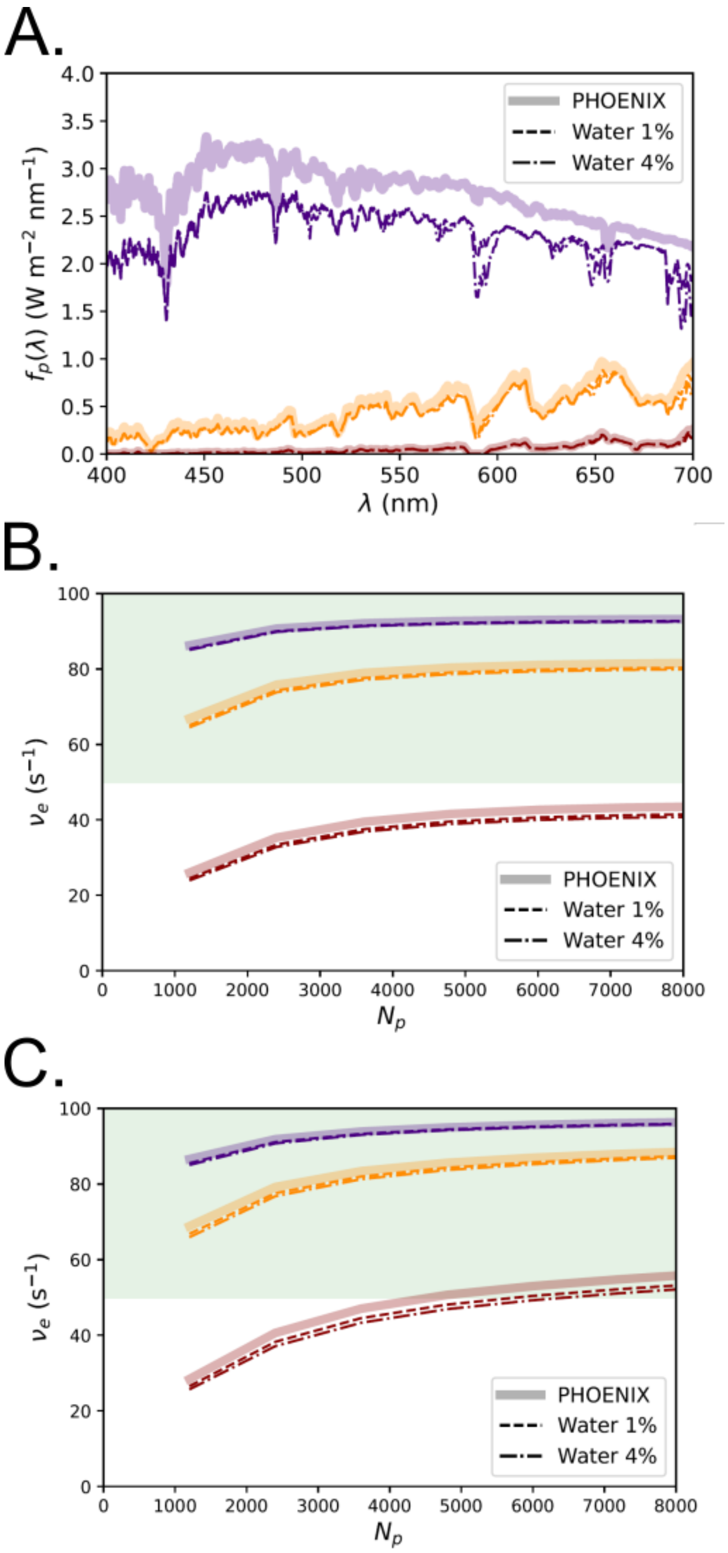

Figure S2: A. The spectral flux for $T_s = 5800$ K (indigo), 3300 K (orange), and 2300 K (dark red), for the unfiltered PHOENIX model (thick, faded line), transmitted through an Earth-like atmosphere with 1% Water (dashed line) and 4% Water. **B.** The electron output for a 'modular' antenna with large LHC sub-units ($N_p^i = 100$) and 12 branches. **C.** The electron output for a 'structured' antenna with large LHC sub-units ($N_p^i = 100$) and 12 branches.

Supplementary References

Villanueva G. L., Smith M. D., Protopapa S., Faggi S., Mandell A.M., 2018, Journal of Quantitative Spectroscopy and Radiative Transfer, 217, 86-104.

Villanueva G. L., Liuzzi G., Faggi S., Protopapa S., Kofman V., Fauchez T., Stone S. W., Mandell A. M., 2022, Fundamentals of the Planetary Spectrum Generator., Provided by the SAO/NASA Astrophysics Data System
